# Signal pathways of melanoma and targeted therapy

**DOI:** 10.1038/s41392-021-00827-6

**Published:** 2021-12-20

**Authors:** Weinan Guo, Huina Wang, Chunying Li

**Affiliations:** Department of Dermatology, Xijing Hospital, Fourth Military Medical University, No. 127 of West Changle Road, 710032 Xi’an, Shaanxi China

**Keywords:** Skin cancer, Drug development

## Abstract

Melanoma is the most lethal skin cancer that originates from the malignant transformation of melanocytes. Although melanoma has long been regarded as a cancerous malignancy with few therapeutic options, increased biological understanding and unprecedented innovations in therapies targeting mutated driver genes and immune checkpoints have substantially improved the prognosis of patients. However, the low response rate and inevitable occurrence of resistance to currently available targeted therapies have posed the obstacle in the path of melanoma management to obtain further amelioration. Therefore, it is necessary to understand the mechanisms underlying melanoma pathogenesis more comprehensively, which might lead to more substantial progress in therapeutic approaches and expand clinical options for melanoma therapy. In this review, we firstly make a brief introduction to melanoma epidemiology, clinical subtypes, risk factors, and current therapies. Then, the signal pathways orchestrating melanoma pathogenesis, including genetic mutations, key transcriptional regulators, epigenetic dysregulations, metabolic reprogramming, crucial metastasis-related signals, tumor-promoting inflammatory pathways, and pro-angiogenic factors, have been systemically reviewed and discussed. Subsequently, we outline current progresses in therapies targeting mutated driver genes and immune checkpoints, as well as the mechanisms underlying the treatment resistance. Finally, the prospects and challenges in the development of melanoma therapy, especially immunotherapy and related ongoing clinical trials, are summarized and discussed.

## Introduction

Melanoma is the most lethal type of skin cancer that originates from the malignant transformation of melanocytes. Actually, melanocytes are of neuroectodermal origin and then migrate extensively to reside throughout the body, including skin, uveal, mucosa, inner ear, and rectum, displaying as highly dendritic cells to manufacture melanin to defend against photodamage.^1^ Due to the relatively wide distribution of melanocytes, melanoma can occur ubiquitously regardless of the anatomical location or the types of organs and tissues. Worldwide, the incidence of melanoma occupies around 1.7% of all newly-diagnosed primary malignant cancers, and patients dying from melanoma account for nearly 0.7% of all cancer mortality.^2^ Of note, the incidence and mortality of melanoma vary among different countries, being relatively high in Australia, New Zealand, Europe, and Northern America, and lowest in Africa. The discrepancy is associated with ethnicity, lifestyle, and genetic background.^2^ The prevalent subtypes and pathogenesis of melanoma in different populations are distinct. White populations with fair skin mainly suffer from cutaneous melanoma, of which the etiology is largely attributed to ultraviolet (UV) exposure. However, pigmented populations from Asia and Africa mainly develop acral and mucosal melanomas at relative lower incidence rates. Trauma and chronic inflammation have been documented as risk factors of acral melanoma, which is supported by the reports that the occurrence of acral melanoma is frequently at the lesions with trauma, infection, and chronic ulcer.^3^ In 2021, there are estimated 106,110 cases of melanoma emerging and 7180 deaths arising from this disease in the United States. Although the mortality rate of melanoma patients is prominently reduced in the past few decades due to early diagnosis, proper screening approaches, improved surgery principle and revolutionary advances of targeted therapy and immunotherapy, the prognosis of patients, in particular those with distant metastasis, remains unoptimistic with the 5-year survival rate around 27%.^4^

**Fig. 1:**
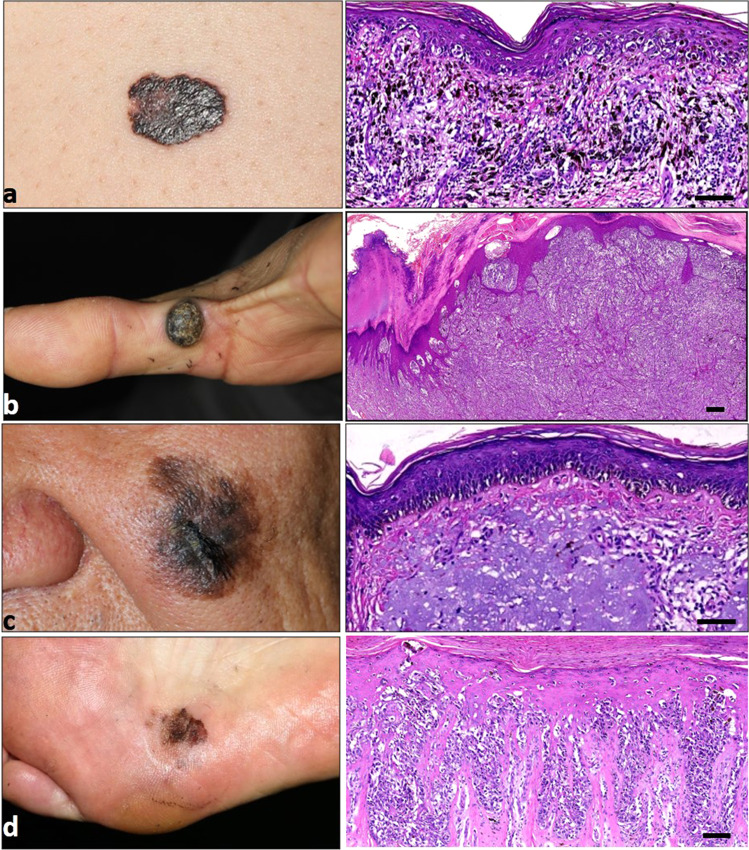
Clinical and corresponding histopathological images of melanomas. Generally, melanomas are classified into four main types according to the histopathological characteristics, namely, superficial spreading melanoma (**a**), nodular melanoma (**b**), lentigo maligna melanoma (**c**), and acral lentiginous melanoma (**d**). The corresponding histopathological image of the same patient is displayed on the right. Scale bar = 100 μm

Clinically, melanoma cases are stratified as the following major subtypes according to their histopathological characteristics, namely, superficial spreading melanoma, nodular melanoma, lentigo maligna melanoma, and acral lentiginous melanoma. To be specific, superficial spreading melanoma usually refers to melanoma in a radial or horizontal growth phase with tumor cells distributing as a nest or solitary units displaying in a pagetoid pattern, whereas nodular melanoma generally occurs in the vertical growth phase. In addition, lentigo maligna melanoma has a significant sign of chronic UV radiation (UVR), and has cells individually distributing alongside the dermal–epidermal junction and skin appendages. Moreover, acral lentiginous melanoma histologically presents as tumor cells in single units along the dermal–epidermal junction and as confluent foci, and commonly occurs at acral sites^5^ (Fig. 1). There are also some other subtype variants defined by clinical or histological characteristics including ocular melanoma, mucosal melanoma, acral melanoma, spitzoid melanoma, and desmoplastic melanoma.

The risk factors of melanoma supported by strong epidemiologic evidence include UVR, multiple moles, family history (a family history/personal history of melanoma), and fair skin, eye, and hair.^1^ Epidemiologic studies by systemic review and meta-analysis have unveiled that intense intermittent UVR exposure through either sunburns or indoor tanning for those before 35 years old, even during childhood, confers the highest risk.^6^ Intense UVR can induce genetic alterations like DNA damage and genetic mutation, reactive oxygen species (ROS) accumulation, and oxidative stress, as well as inflammatory responses involving macrophages and neutrophils infiltration, which are related to malignant switch of melanocytes.^7–10^ Moreover, the phenotypic characteristics of fair skin, eye, and hair indicate the insufficient ability of melanocytes to generate eumelanin that have stronger photoprotective capacity than pheomelanin, rendering individuals more vulnerable to sun exposure and other environmental stress on the skin.^11^ Of note, UV exposure accounts for two distinct etiological mechanisms for melanoma pathogenesis.^12^ On one hand, early sun exposure and proneness to nevi tend to induce melanoma carcinogenesis driven by *BRAF* mutation. Patients with these risk factors are usually diagnosed at a young age and generally display superficially spreading melanoma on the trunk. On the other, chronic sun exposure often leads to melanoma harboring *NRAS* mutation, without any involvement of nevi proneness. Apart from environmental UVR and some phenotypic characteristics in individuals that are associated with the carcinogenesis of cutaneous melanoma, some other factors are documented to be associated with non-cutaneous melanoma, especially trauma and chronic inflammation for acral melanoma. A previous study conducted by our group that involves 685 Chinese patients with melanoma has revealed a prominent correlation between acral melanoma and the history of trauma on the lesion.^13^ In addition, some reports also indicated the generation of acral melanoma after trauma or from lesions of infection and chronic ulcer.^14–16^ The pro-tumorigenic effect of trauma and chronic inflammation might result from the increased cytokines and ROS that can induce genetic instability or activate oncogenic pathways in melanocytes.^17^

**Fig. 2:**
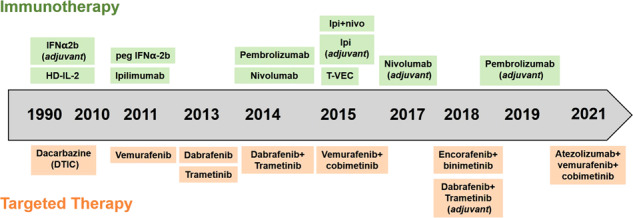
Timeline for FDA-approved therapies for metastatic melanoma. HD high-dose, Ipi Ipilimumab, T-VEC talimogene laherparepvec

The therapeutic approaches of melanoma undergo a dramatic evolution in the past few decades due to the progress in the understanding of melanoma pathogenesis and thereby revolutionary advances of targeted therapies that specifically intervene mutant driver genes and immune checkpoints. Historically, there were only dacarbazine chemotherapy and high-dose interleukin-2 (IL-2) approved by Food and Drug Administration (FDA) as treatment agents for metastatic melanoma before 2010. Interferon-α2b (IFN-α2b) was also employed as adjuvant agent, whereas the usage was largely limited due to the frequent occurrence of severe adverse effects.^1,18^ Since ten years ago, a series of therapeutic agents and combinatorial approaches have been approved by FDA, including immunotherapy (single-agent ipilimumab, nivolumab, pembrolizumab, and combination of ipilimumab and nivolumab), targeted therapy (single-agent vemurafenib and dabrafenib, combinations of dabrafenib plus trametinib, vemurafenib plus cobimetinib, and encorafenib plus binimetinib) as well as one intralesional modified oncolytic herpes virus talimogene laherparepvec (T-VEC) (Fig. 2 and Table 1).^1,18–21^ The above-mentioned therapeutic approaches have gained evident and encouraging responses in treating patients with advanced melanoma and some of them have also been approved in the adjuvant setting. Compared to 10 years ago, the 5-year survival has gained considerable improvement from <5% to around 30% in patients with advanced melanoma who accept the combination of BRAF inhibitor and MEK inhibitor or single anti-PD-1 antibody.^21–23^ Although current therapies have revolutionized the standard of management for patients with advanced melanomas, low response rate and inevitable occurrence of treatment resistance retard forward improvement of therapeutic outcome.^24^ Therefore, it is necessary to understand the molecular mechanisms underlying melanoma pathogenesis more comprehensively, which might lead to the innovations of more applicable therapeutic approaches and provide additional clinical options for melanoma therapy.

**Table 1:** FDA-approved therapies for melanoma

Agent	Mechanism	Type	Indications	Time
Dacarbazine	Alkylating agent	Chemotherapy	Metastatic melanoma	1975
IL-2	Cytokine	Immunotherapy	Metastatic melanoma	1998
IFN-alfa-2b	Cytokine	Immunotherapy	Adjuvant therapy for high risk of recurrent melanoma	1995
Pegylated IFN-alfa-2b	Cytokine	Immunotherapy	Resected stage III melanoma	2011
T-VEC	Oncolytic virus	Immunotherapy	For the local treatment of unresectable cutaneous, subcutaneous, and nodal lesions in patients with melanoma recurrent after initial surgery	2015
Vemurafenib	BRAF inhibitor	Targeted therapy	Unresectable/metastatic melanoma with BRAF V600E/K mutation	2011
Cobimetinib	MEK inhibitor	Targeted therapy	Unresectable/metastatic melanoma with BRAF V600E/K mutation	2015
Dabrafenib	BRAF inhibitor	Targeted therapy	Unresectable/metastatic melanoma with BRAF V600E/K mutation	2013
Trametinib	MEK inhibitor	Targeted therapy	Unresectable/metastatic melanoma with BRAF V600E/K mutation	2013
Dabrafenib + tremetinib	BRAF inhibitor + MEK inhibitor	Targeted therapy	Unresectable/metastatic melanoma with BRAF V600E/K mutation Adjuvant treatment of patients with melanoma with BRAF V600E/K mutation	2015
Vemurafenib + cobimetinib	BRAF inhibitor + MEK inhibitor	Targeted therapy	Unresectable/metastatic melanoma with BRAF V600E/K mutation	2015
Encorafenib + binimetinib	BRAF inhibitor + MEK inhibitor	Targeted therapy	Unresectable/metastatic melanoma with BRAF V600E/K mutation	2018
Ipilimumab	Anti-CTLA-4 antibody	Immunotherapy	Unresectable/metastatic melanoma Adjuvant treatment of resected stage III melanoma	2011
Nivolumab	Anti-PD-1 antibody	Immunotherapy	Unresectable/metastatic melanoma Adjuvant treatment of resected stage III melanoma	2014
Pembrolizumab	Anti-PD-1 antibody	Immunotherapy	Unresectable/metastatic melanoma Adjuvant treatment of resected stage III melanoma	2014
Nivolumab + ipilimuab	Anti-CTLA-4 antibody + anti-PD-1 antibody	Immunotherapy	Unresectable/metastatic melanoma	2015
Atezolumab + vemurafenib + cobimetinib	Anti-PD-L1 antibody + BRAF inhibior + MEK inhibitor	Combination therapy	Unresectable or metastatic melanoma with BRAF V600E/K mutation	2020

In this review, we systemically summarized the signal pathways driving melanoma pathogenesis, including the main mutated driver genes and signals, key transcriptional factors and downstream molecular biology, epigenetic modification, metabolic reprogramming, crucial metastasis-related signals, and tumor-promoting inflammatory pathways and pro-angiogenic factors. Then, current progress in therapies targeting mutant driver genes and immune checkpoints, and novel combined therapeutic approaches were introduced, with some discussions about the mechanism underlying treatment resistance. Finally, the ongoing clinical trials and future perspectives of clinical advances were concluded.

## Signal pathways driving melanoma pathogenesis

### Mutated driver genes and downstream signal pathways

In the past few decades, the methods to dig into the genomic alterations in driving melanoma carcinogenesis have evolved and helped to gain more and more encouraging insights. Multiple mutated driver genes have been identified and are enriched in various signal pathways that are pivotal contributors to melanoma carcinogenesis and development, including mitogen-activated protein kinase (MAPK) pathway, protein kinase B (AKT) pathway, cell-cycle regulation pathway, pigmentation-related pathway, p53 pathway, epigenetic factors, and some other pathways.

MAPK pathway is frequently activated in cancer to enable tumor cell rapid proliferation. In response to extracellular binding of growth factors to receptor tyrosine kinases (RTKs), intracellular sequential activation of Ras, Raf, MEK, and ERK occurs to regulate a plenty of oncogenic biological activities. For melanoma, mutations in the key signal components, including *BRAF*, *NRAS*, *NF1,* and *KIT*, are responsible for the hyper-activation of the MAPK pathway. BRAF is a serine/threonine kinase belonging to Raf family and implicated in the signal transduction in the MAPK pathway. In 2002, a genome-wide screening discovered a point mutation of *BRAF* occurring more frequently in melanoma than other types of solid tumors. The substitution from valine to glutamic acid at codon 600 (V600E), which leads to constitutive activation of the kinase activity of BRAF protein and downstream MAPK pathway, can be detectable in ~50% of melanomas.^25^ Other variants including V600K, V600D, and V600R occupy around 12%, 5%, and 1% of *BRAF* mutations, respectively.^26,27^ The presence of *BRAF* mutations has great potential in predicting an unfavorable prognosis in melanoma patients.^28,29^ In other subtypes of melanoma like acral melanoma and mucosal melanoma, the incidence of *BRAF* mutation is around 20% and 6%, which is much lower than that in cutaneous melanoma.^30,31^ NRAS is a small GTP-binding protein belonging to Ras family and transduces upstream RTK activation to promote the activity of downstream Raf. The mutations of *NRAS* usually occur at G12, G13, and Q61 sites, and are found in around 25% of cases of melanomas.^32^ Compared to *BRAF*^V600E^ mutation which can be effectively targeted by some agents like vemurafenib and dabrafenib, therapeutic options for melanoma harboring *NRAS* mutations lag behind. A recent study has identified STK19 as a novel NRAS activator by enhancing its phosphorylation and binding to downstream effectors. Therefore, the blockade of STK19 kinase activity might be a promising strategy to treat melanomas harboring *NRAS* mutations.^33^ Moreover, through the technology of whole-exome sequencing, *NF1*, that encodes a negative regulator of RAS, has been identified as the third most frequently mutated gene in melanoma after *BRAF* and *NRAS.*^34,35^ The loss of *NF1* or inactivating *NF1* mutation are present in 46% of melanomas expressing wild-type *BRAF* and *RAS*, which leads to constitute activation of Ras by lessening its intrinsic GTPase activity and induces hyper-activation of MAPK pathway.^36^ What’s more, *KIT* mutation and amplification, that cause receptor dimerization, auto-phosphorylation of tyrosine residues, and the activation of downstream oncogenic pathways, are mainly found in mucosal and acral melanomas (10–20% of these types).^37^

**Fig. 3:**
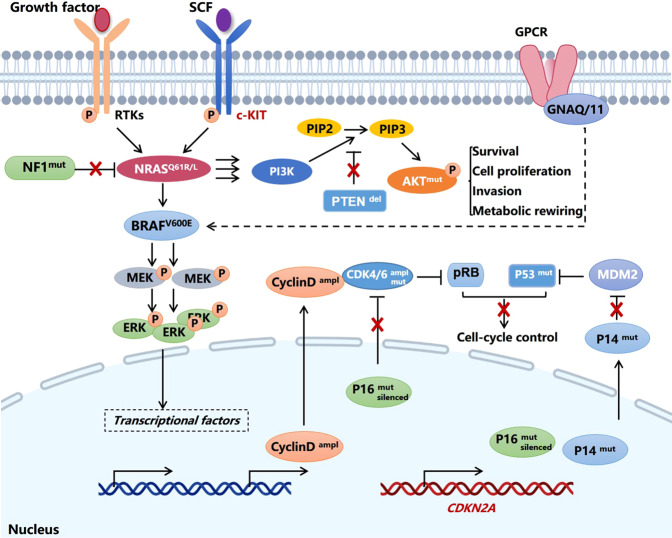
Mutated driver genes and downstream signal pathways in melanoma. Ampl amplification, CDK cyclin-dependent kinase, Del deletion, GPCR G protein-coupled receptor, Mut mutation, P (in a pink circle) phosphate, p14^ARF^ and p16^INK4A^ splice variant encoded by *CDKN2A* gene, PIP2 phosphatidylinositol-(4,5)-bisphosphate, PIP3 phosphatidylinositol-(3,4,5)-trisphosphate, PTEN phosphatidylinositol‑3,4,5‑trisphosphate 3‑phosphatase, and dual-specificity protein phosphatase, RB retinoblastoma-associated protein, RTK receptor tyrosine kinase, SCF stem cell factor

Uncontrolled cell-cycle progression is a hallmark characteristic of melanoma development, and multiple components in this process are found to be mutated, including cyclin-dependent kinase inhibitor 2A (CDKN2A), retinoblastoma-associated protein (RB), Cyclin D1, and cyclin-dependent kinase 4/6 (CDK4/6). To be specific, the risk loci in *CDKN2A* is identified in about 40% of familial melanomas. Physiologically, *CDKN2A* encodes two proteins p16^Ink4a^ and p14^Arf^ through distinct translational programs. Wild-type p16^Ink4a^ restrains cell-cycle progression by abrogating cyclin-dependent kinase 4 (CDK4) or CDK6-mediated phosphorylation and inactivation of RB. In addition, wild-type p14^Arf^ can prevent E3 ubiquitin ligase MDM2-mediated degradation of p53 to control cell cycle. Therefore, germline mutation of *CDKN2A* induces the functional deficiency of both p16^Ink4a^ and p14^Arf^, which leads to uncontrolled cell-cycle progression by impairing the function of downstream RB1 and p53, respectively (Fig. 3). Besides, the aberrations of *CCND1* and CDK4/6 pathways have been documented to occur more frequently in acral melanoma than cutaneous melanoma, with the incidence being around 40% and 80%, respectively.^38,39^ The amplification rates of *CDK4* and *CCND1* are also frequently found in mucosal melanoma, with the incidence being 47.0% and 27.7%, respectively.^40^ Therefore, targeting the CDK4/6 pathway by specific inhibitor is hopeful to bring encouraging outcome for the treatment of acral melanoma and mucosal melanoma.

The dysregulated activation of the AKT pathway occurs in around 70% of total melanomas, which is the result of *AKT3* amplification and *PTEN* loss by epigenetic silencing or deletion as previously described.^41,42^ Generally, the activation of the AKT pathway is initiated by activated phosphoinositide 3-kinase (PI3K) after the stimulation by exogenous growth factors, followed by increased generation of the second messenger phosphatidylinositol-3,4,5-trisphosphate (PIP3) that can promote the translocation of AKT to the plasma membrane for its subsequent phosphorylation and activation. Since that intracellular level of PIP3 is negatively regulated by the phosphatase PTEN, the functional deficiency of PTEN can induce the upregulation of PIP3 level and promote AKT activation.^43^ It has been documented that the predominant AKT isoform in melanoma is AKT3. SiRNA transfection targeting AKT3 or overexpression of PTEN can effectively suppress AKT3 activity to reduce the tumorigenic potential of melanoma cells.^42^ Therefore, the hyper-activation of AKT pathway is a pivotal oncogenic event for melanoma carcinogenesis and development.

As for sporadic cases of melanomas that account for around 90% of total melanomas, they are mostly driven by the mutations in genes implicated in pigmentation process like *MC1R* (encoding melanocortin-1 receptor), *TYR* (encoding tyrosinase), *TYRP1* (encoding tyrosinase-related protein-1), *PAX3* (encoding paired box 3), *EDNRB* (encoding endothelin receptor type B), *ASIP* (encoding agouti signaling protein), *OCA2* (encoding oculocutaneous albinism II), *SLC45A2* (encoding solute carrier family 45 member 2) and *SOX10* (encoding SRY-box transcription factor 10), which dictates the causal relationship between UV radiation and increased risk of melanoma.^44–46^ In response to the stimulation of α‑melanocyte-stimulating hormone (α-MSH), MC1R can be activated to transduce downstream signals to induce the expression of microphthalmia-associated transcription factor (MITF), a master regulator of the generation of melanin.^47^ TYR and TYRP1 are the main downstream targets of MITF implicated in melanin production, whereas SOX10 and PAX3 are a canonical melanocytic lineage-specific transcriptional factor of MITF.^48^ The functional deficiency of the above-mentioned genes causes unbalanced production of UV‑protective eumelanin and less-protective pheomelanin in melanocytes,^49,50^ thus contributing to melanoma carcinogenesis in highly-risky individuals encountering environmental insults.

Apart from MAPK pathway, cell-cycle regulation pathway, AKT pathway, and pigmentation-related pathway, genetic mutations in some other pathways are also implicated in the carcinogenesis of melanoma. The activating mutations in *NOTCH2*, *CTNNB1* can lead to aberrant activation of Notch and Wnt pathway respectively to facilitate the pathogenesis of melanomas of different subtypes, including cutaneous, acral, and mucosal melanomas.^51–53^ In addition, *GNAQ*/*GNA11* mutations are the major genetic drivers in uveal melanoma with the incidence of 80–90%,^54,55^ which contribute to the hyper-activation of downstream MAPK pathway. What’s more, mutations in *TP53*, *ARID1B*, *ARID2,* and *TERT* and the amplification of *MDM2* have also been identified in melanoma, which are involved in the regulation of p53 pathway, SWI/SNF chromatin remodeling complex, and telomerase activity respectively.^31,56–59^

Taken together, the above-mentioned high-frequency mutations depict the framework of the melanoma mutational landscape, and more importantly, provide multiple druggable targets for precisely intervening specific signaling pathways. Although the mutational landscape in cutaneous melanoma has been extensively investigated, the genetic aberrations of acral melanoma and mucosal melanoma are far from understood. Further investigations are needed to clarify the mutational landscape of non-cutaneous melanoma in the future.

### Key transcriptional signal pathways implicated in melanoma development

Melanoma originates from epidermal melanocytes and shares many molecular similarities with melanocyte precursors, indicating that the developmental program of melanocytes, especially the transcriptional regulation program, is utilized by melanoma cells to facilitate tumor progression.^60^ In fact, multiple key transcriptional factors and signal pathways responsible for the formation of melanocytic lineage, including SOX10, MITF, Notch, and Wnt-β-catenin, are greatly implicated in the malignant characteristics of melanoma cells.

**Fig. 4:**
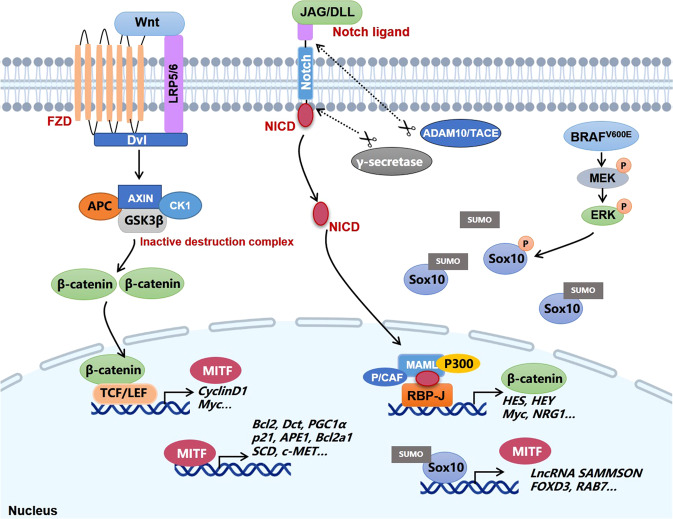
Key transcriptional factors and signal pathways in melanoma. ADAM10 ADAM metallopeptidase domain 10, APC adenomatosis polyposis coli, DLL delta-like canonical Notch ligand, DVL Disheveled segment polarity protein, FZD frizzled class receptor, GSK3β Glycogen synthase kinase 3β, JAG jagged canonical Notch ligand, LEF-1 lymphoid enhancer binding factor 1, LRP5/6 low density lipoprotein receptor-related protein 5/6, MAML mastermind-like transcriptional coactivator, NICD Notch intracellular domain, P/CAF P300/CBP-associated factor, RBPJ recombination signal binding protein for immunoglobulin κ J region, TACE TNFα-converting enzyme

SOX10 is a neural crest transcription factor essential for the initiation and development of Schwann cells and melanocytes. It has been identified as a specific marker with relatively high sensitivity and specificity for the diagnosis of melanocytic and Schwannian tumors, including metastatic melanoma occurring in sentinel lymph nodes.^61–63^ Preliminary studies have demonstrated that haplo-insufficiency of SOX10 inhibits mutant *NRAS*-driven formation of congenital nevus and melanoma in transgenic mice model. Genetic knockdown of SOX10 expression robustly abolishes the proliferative and migratory capacity of melanoma cells in vitro and the growth of melanoma in vivo, implicating the fundamental role played by SOX10 in maintaining melanoma cell survival.^64–66^ As a transcriptional factor, SOX10 activates various targets like MITF, long non-coding RNA (lncRNA) SAMMSON, forkhead box D3 (FOXD3), and RAB7 to regulate cell proliferation, mitochondrial function, and endolysosomal pathway, therefore affecting various biological activities in melanoma^67–70^ (Fig. 4). Meanwhile, SOX10 expression is under the control of multiple species-conserved regulatory sequences in the upstream region of its encoding gene that can be bound by several other transcriptional factors.^71^ Besides, multiple post-translational modification paradigms like sumoylation, ubiquitination, and phosphorylation also participate in the regulation of SOX10 transcriptional activity and protein stability.^67,72–74^ In a previous study on the mechanism underlying vemurafenib resistance, using a chromatin-regulator-focused shRNA library, the authors identified the loss of SOX10 expression as a crucial cause of the resistance to targeted therapy via the activation of transforming growth factor-β (TGF-β) signaling and subsequent upregulation of epithelial growth factor receptor (EGFR) and platelet-derived growth factor receptor β (PDGFRβ), linking SOX10 to a slow-growth resistance phenotype.^75,76^ However, two recent reports have demonstrated that the depletion of SOX10 expression or its downstream SAMMSON sensitizes *BRAF*-mutant melanoma to MAPK-inhibition agents,^67,77^ supporting that SOX10 upregulation contrarily triggers the resistance to targeted therapy. The above-mentioned paradoxical role of SOX10 in the resistance to MAPK inhibition-targeted therapy can be associated with the different characteristics between adaptive resistance and acquired resistance occurring at different phases after treatment. To be specific, during the early phase of inhibitor treatment, adaptive response would be readjusted to enable tumor cell survival, and increased transcriptional activity of SOX10 is responsible for the upregulation of two cyto-protective factors FOXD3 and SAMMSON, rendering adaptive resistance. Nevertheless, mutational acquired resistance will prevail after a long-term inhibitor treatment, with the upregulation of multiple receptor tyrosine kinases (RTKs) greatly involved in. The downregulation of SOX10 predominantly tends to induce the senescent phenotype and compensatory reactivation of receptor tyrosine kinases in this phase, rather than to suppress tumor cell survival.^76–78^ Therefore, the biological effect of SOX10 in melanoma targeted therapy is context-dependent, raising the notion that the intervention of SOX10 expression for overcoming resistance to targeted therapy should take the phases and paradigms of drug resistance into consideration.

MITF is an extensively studied melanocytic lineage-specific transcriptional factor, which coordinates many signal pathways of melanoma biology including cell proliferation, survival, metastasis, metabolism, phenotypic plasticity, antitumor immunity, and therapeutic resistance.^79^ Initial investigations regarding the role of MITF in melanoma cell proliferation had obtained confusing conclusions,^80,81^ which were then largely reconciled by the “rheostat model” proposed by Carreira et al. To be specific, low MITF expression is associated with slow-cycling state characterized by the upregulation of cell-cycle inhibitor and the potentiation of invasive and metastatic capacity. In contrast, moderate MITF expression induces phenotype switch from invasive to proliferative state, endowing melanoma cells with a stronger growth advantage. However, when MITF expression is further upregulated, a differentiation-associated G1 arrest would be re-established.^82^ This model suggests that the biological function of MITF is highly correlated with its activity level. Since that MITF is an integrated transcriptional factor governing the expressions of a plenty of target genes implicated in cell-cycle progression (CDK2, CCND1, p21, p16, and p27), cell differentiation (TYR, TYPR1, DCT, RAB27, MYO5a), and invasive capacity (GMPR, DIAPH1),^79^ the cellular phenotype regulated by MITF is possibly attributed to the mixture of dysregulated molecules involved in distinct biological aspects. The transcription of genes responsible for cell-cycle arrest and invasion predominates when MITF is expressed at a low level, whereas moderate to high MITF expression mainly potentiates downstream targets that facilitate cell proliferation and de-differentiation. Different from the paradoxical role of MITF in cell proliferation, many reports have provided compelling evidence to support the essential role of MITF in melanoma cell survival. Through the regulation of a series of transcriptional targets like Bcl2, Bcl2a1, ML-IAP, HIF1α, c-MET, APE1, p21, and BRAC1, MITF plays its pro-survival role under normal or stressful conditions by regulating oxidative stress, cell senescence, DNA damage repair, and oncogenic pathways (Fig. 4).^83–88^ While the correlation between low MITF expression and high invasive capacity of melanoma cell has long been observed, the underlying mechanism is only recently elucidated by Bianchi-Smiraglia et al. Their study has disclosed that MITF suppresses the invasive capacity by reducing intracellular GTP pools and subsequent amounts of active (GTP-bound) RAC1, RHO-A, and RHO-C, mainly via the transcriptional regulation of guanosine monophosphate reductase (GMPR).^89^ In addition, downregulation of MITF mediates the facilitative role of signal transducer and activator of transcription 3 (STAT3) in melanoma metastasis under the transcriptional control of cAMP-response element-binding protein (CREB),^90^ further confirming the enhanced invasive phenotype in MITF^low^ melanomas. The regulation of melanoma cell phenotypic plasticity by MITF is also attributed to the alteration of cell metabolism. MITF is recently discovered to be the lineage-restricted transcriptional activator of the key lipogenic enzyme stearoyl-CoA desaturase (SCD). By promoting the conversion from saturated fatty acids to mono-unsaturated fatty acids, SCD upregulation is required for highly-expressed MITF-driven melanoma cell proliferation. In contrast, the suppression of SCD in MITF^low^ melanoma cells accentuates the invasive and metastatic capacity by activating inflammation-related signaling and induces de-differentiation state.^91^ In addition to the above-mentioned biological effects, MITF expression can also dictate the sensitivity of *BRAF*-mutant melanoma to MAPK- inhibition-targeted therapy, though the role is paradoxical in terms of the phase of drug resistance establishment. While low MITF expression is highly associated with intrinsic/acquired resistance to targeted therapy and especially renders increased resistance in aged microenvironment,^92,93^ the upregulation of MITF is considered as a protective factor to defend against BRAF inhibitor-induced cell apoptosis and mediates adaptive resistance within a relative short duration after treatment.^78,94^ Based on this, a drug-repositioning screening has identified the HIV1-protease inhibitor nelfinavir as a promising drug to overcome the adaptive resistance to MAPK pathway inhibitors via the downregulation of MITF expression.^95^ Therefore, the intervention of MITF expression to modulate the outcome of targeted therapy should also take the phase of drug resistance establishment into account.

Notch signal pathway has been documented as a cardinal signal pathway implicated in stem cell self-renewal, cell differentiation, and cell fate decisions in many organs.^96^ The oral administration of a γ-secretase inhibitor (GSI) that can block Notch signal in mice has been reported to impair hair pigmentation, even 20 weeks after discontinuing the treatment, indicating that Notch is essential for maintaining the homeostasis of melanocyte.^96^ Compared with normal melanocytes, the expression and activity of Notch are significantly higher in melanoma. Melanocytes transfected with truncated Notch transgene construct (N(IC)) containing enhanced Notch activity display augmented cell proliferation and malignant characteristics similar to melanoma.^97^ The upregulation of Notch in melanoma is the result of both intracellular AKT pathway activation and the presence of tumor microenvironment factors like hypoxia.^98^ As a cardinal driver of cancer pathogenesis, Notch can exert its oncogenic role in melanoma via the activation of MAPK pathway, the upregulation of N-cadherin expression, and the potentiation of β-catenin signaling,^99,100^ indicating that it acts as a nexus node coordinating multiple carcinogenic signals. Supplementary to intrinsic effect on tumor cell behavior, Notch activation is also highly associated with angiogenesis, and participates in the crosstalk between tumor cells and endothelial cells to facilitate tumor migration as well.^101,102^ In particular, sustained Notch1 activation leads to the senescence of endothelial cells and increase of vascular cell-adhesion molecule 1 (VCAM1) expression, thereby contributing to neutrophil infiltration, tumor cell adhesion to endothelium, intravasation, lung colonization, and postsurgical metastasis.^103^ Moreover, a recent study demonstrates that the activation of Notch renders the resistance of *BRAF*-mutant melanoma cells to MEK inhibitors like Cobimetinib.^104^ Therefore, targeting Notch can be exploited to not only restrain the progression of melanoma, but also increase the efficacy of targeted therapy.

Wnt signal is an evolutionarily conserved pathway implicated in embryonic development, tissue regeneration, and cell homeostasis. Previously, the notification of the importance of Wnt in melanocyte development comes from the observation of the absence of melanoblasts in Wnt-deficient mice.^105^ Moreover, the activation of β-catenin downstream Wnt greatly contributes to the cell fate decision from the loss of glial derivatives to the expansion of melanocytes, further supporting the fundamental role of Wnt-β-catenin in melanocyte development. Wnt signaling has two main types of pathways, namely, β-catenin-dependent pathway (canonical) and β-catenin-independent pathway (non-canonical), with their function in melanoma emphasizing cell proliferation and cell polarity/migration respectively.^106^ Takeda et al. has firstly reported that MITF is activated by canonical Wnt-β-catenin signaling via LEF-1^107^ (Fig. 4), which is then proved to be required for the pro-proliferative effect of Wnt in melanoma.^108^ Additional evidence supports the conclusion that the oncogenic role of the Wnt-β-catenin pathway is mediated by the suppression of p16 and the overcome of oncogene-induced senescence. Therefore, co-operation of *NRAS* mutation and Wnt-β-catenin activation can lead to melanoma formation with high penetrance and short latency in mice.^109^ Later on, the role of Wnt in melanoma metastasis receives more attention. Damsky et al. has provided evidence that β-catenin is the central mediator of tumor metastasis to lymph node and lung in established melanoma transgenic mice model induced by both *BRAF* mutation and *PTEN* deficiency.^110^ Moreover, Wnt3a derived from tumor-infiltrating fibroblast leads to β-catenin activation in melanoma cell, which diminishes tumor cell adhesion and enhances migration to form liver metastasis.^111^ Alternative Wnt ligands like Wnt5a derived from myeloid-derived suppressor cells (MDSC) and Wnt11 also participate in melanoma metastasis.^112,113^ However, the exact role of Wnt-β-catenin signal in melanoma development remains controversial even with extensive investigations, namely, β-catenin cannot be fully defined as an oncogene according to available results.^114–116^ In particular, nuclear β-catenin expression has been previously unveiled to be downregulated during melanoma progression.^117,118^ Moreover, a recent study has proved that temporal activation of Wnt/β-catenin signal is sufficient to suppress SOX10 expression via the proteasome degradation and therefore blocks the growth of melanoma.^119^ These reports indicate the tumor-suppressive role of Wnt/β-catenin in melanoma. Therefore, more investigations are needed to forwardly clarify the exact role of Wnt in different contexts, distinct biological activities and heterogeneous genetic backgrounds in melanoma.

In aggregate, the above-mentioned key transcriptional programs driven by SOX10, MITF, Notch, and Wnt-β-catenin signals that share molecular similarities with melanocyte precursors contribute greatly to the malignant switch from melanocyte to melanoma. Of note, these factors that dictate the plasticity and differentiation state of melanoma cells are also decisive for the efficacy of targeted therapy. To overcome the de-differentiation characteristic of tumor cell might be a useful strategy to improve the outcome of patients that receive MAPK inhibition-targeted therapy.^120^

### Epigenetic alterations and the downstream signal pathways

Aside from genetic and transcriptional modulation, epigenetics is emerging as another crucial regulatory paradigm of melanoma biology and signal pathways. Epigenetic modification refers to heritable changes in gene expression without an alteration in the genome sequence, of which the core mechanism is the covalent modifications of either histone tails or nucleosome complexes that can re-shape chromatin structure and modulate gene expression.^121^ DNA methylation, histone modification, non-coding RNA, and newly-discovered N^6^-methyladenosine (m^6^A) RNA methylation are the main types of epigenetic modification, and their dysregulations are highly correlated with melanoma development.^122^

**Fig. 5:**
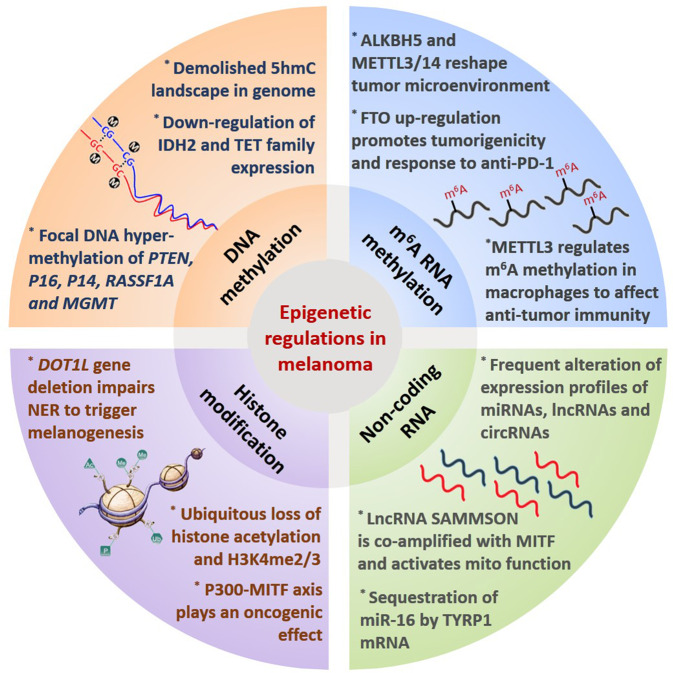
Epigenetic regulation in melanoma. Main paradigms of epigenetic modification and representative effects on cancer biology in melanoma

As the most intensively investigated epigenetic modification in cancer,^122^ DNA methylation involves the addition of a methyl group to the 5 position of cytosine by DNA methyltransferase to form 5-methylcytosine (5-mC). This process is dynamically regulated by DNA methyltransferases (DNMTs) that transfer the methyl group to the cytosine residue and ten-eleven translocation (TET) family that indirectly promotes DNA de-methylation via the oxidative catalysis of 5-mC to form 5-hmC.^123–125^ Lian et al. has discovered that loss of 5-hmC triggered by the downregulation of isocitrate dehydrogenase 2 (IDH2) and TET family members is an epigenetic hallmark of melanoma progression, with potent diagnostic and prognostic implications. Re-establishment of 5-hmC landscape is capable of suppressing melanoma growth and improving the survival of the preclinical mice model.^126^ In addition to the general downregulation of 5-hmC content, focal DNA hyper-methylation of the promoters of some specific tumor suppressors has also been well illustrated in melanoma, in particular *PTEN*, *P16*^INK4A^, *P14*^ARF^, *RASSF1A,* and *MGMT* (occurring in ~60%, 30%, 80%, 55%, and 30% of melanomas, respectively)^127–134^ (Fig. 5), which is associated with functional deficiency of these genes during melanoma progression. Besides, a series of alternative genes have been identified to be differentially-methylated at the promoter region between melanoma and benign nevi by genome-wide promoter methylation analysis.^135–137^ These genes are generally enriched in signal pathways orchestrating cell differentiation, immune-related function, epithelial-to-mesenchymal transition, PI3K/mTOR signaling, metastasis, and cellular metabolism that are all hallmark characteristics of cancer biology.^138,139^

Chromatin is dynamically switched between two states, namely, dense heterochromatin state of deficient transcriptional activity and relaxed euchromatin state with active transcriptional capacity.^140^ Histone modifications refer to a class of post-translational modifications (PTMs) typically occurring on the N-terminal “tails” of histones, which can shape the structure of chromatin and thereby modulate the accessibility of DNA for gene transcription or DNA damage repair.^141,142^ The histone PTMs typically include acetylation, methylation, phosphorylation, and ubiquitination.^143^ To be specific, histone acetylation is defined by the addition of acetyl-CoA to lysine residues that can be reversely regulated by histone acetyltransferases (HATs) and histone deacetylases (HDACs).^144^ Besides, histone methylation frequently occurs at lysine or arginine residues at specific sites on histones H3 and H4 that can also be dynamically regulated by lysine-specific demethylase (LSD) and multiple methyltransferases.^145,146^ Recently, systematic epigenomic profiling of 35 epigenetic modifications and transcriptomic analysis has revealed the ubiquitous loss of histone acetylations and H3K4me2/3 on regulatory regions proximal to specific cancer-regulatory genes in tumor-driving pathways in melanoma (Fig. 5). In parallel, the expressions of HATs and HDACs in tumorigenic transition are also ubiquitously dysregulated.^147^ On this basis, the restoration of histone acetylation with HDAC inhibitors has prominent tumor suppressor potential,^147,148^ and can also synergistically increase the efficacy of radiotherapy, MAPK pathway-targeted therapy, and immunotherapy via the regulation of DNA damage repair, intracellular ROS generation, and programmed death ligand-1(PD-L1) expression, respectively.^149–152^ Some novel selective inhibitors of HDAC families have been developed and exploited in first-in-human study to testify the therapeutic effect on patients with refractory solid tumors including melanoma.^153^ Aside from HDAC, investigations regarding histone acetyltransferases demonstrate the pivotal role of P300 in melanoma development. Selective pharmacological inhibition of P300 displays a prominent tumor-suppressive effect.^154^ The expression of MITF can be a promising predictor of the therapeutic vulnerability to P300 inhibition^155,156^ (Fig. 5). Of note, the activation of P300 also contributes to the resistance to BRAF-targeted therapy by activating ERK signal and mitochondrial oxidative phosphorylation,^157,158^ highlighting targeting P300 as a valuable synergistic therapeutic approach to sensitize melanoma cells toward MAPK pathway inhibition.

Supplementary to histone acetylation, histone methylation also impacts chromatin condensation and gene transcription to regulate melanoma biology,^145^ with some enzyme families like SET domain bifurcated 1 (SETDB1), disruptor of telomeric silencing 1-like proteins (Dot1L), enhancer of Zeste homolog 2 (EZH2) and LSD1 implicated in. SETDB1 is responsible for the methylation of histone H3 on lysine 9 (H3K9) and is recurrently amplified in melanoma to play an oncogenic role.^159,160^ Metabolic reprogramming endows melanoma cells with increased histone H3 trimethylation and paralleled higher metastatic capacity that can be reversed by the pharmacological inhibition of SETDB1.^161^ Different from SETDB1, *DOT1L* gene is located in a frequently deleted region and undergoes somatic mutation that compromises its methyltransferase enzyme activity which leads to reduced H3K79 methylation. The loss of function of Dot1L accelerates UVR-triggered melanoma development by impairing the recruitment of nucleotide excision repair (NER) machinery XPC and hindering DNA damage repair^162^ (Fig. 5), indicating that Dot1L is a protector against melanomagenesis. Moreover, the expression of another histone methyltransferases EZH2 is upregulated in melanoma, which promotes H3K27 trimethylation to silence multiple tumor suppressors.^163,164^ Deficiency of cilium construction and peroxisome proliferator-activated receptor γ coactivator-1 (PGC1α) resulting from EZH2 upregulation significantly activates both Wnt/β-catenin and Yes-associated protein (YAP) signals to drive melanoma metastasis.^165,166^ Notably, the dysregulation of EZH2 is also greatly implicated in the resistance to immunotherapy by downregulating antigen presentation, IFN-γ gene signature, and lymphocytes infiltration,^167–169^ implying that EZH2 might be versatile player in melanoma pathogenesis. What’s more, it has been reported that two different types of H3K9 demethylases, LSD1 and JMJD2C, abolish oncogenic *RAS*- or *BRAF*-induced senescence by promoting the expression of E2F target genes, cooperatively driving melanomagenesis. Specific inhibition of highly-expressed H3K9-active demethylases restores oncogene-induced senescence and suppresses melanoma development.^170^ In addition, the ablation of LSD1 enhances tumor immunogenicity via the upregulation of endogenous retroviral element (ERV) transcripts and the downregulation of RNA-induced silencing complex. This mechanism is also responsible for the diminished resistance to anti-PD-1 immunotherapy in melanoma, suggesting LSD1 as a promising immunotherapy target.^171^ It seems that H3K9 methylation regulated by SETDB1, LSD1, and JMJD2C plays contrary roles in melanoma carcinogenesis and development respectively. While the restoration of H3K9 methylation by targeting LSD1 and JMJD2C abrogates the transition from melanocyte to melanoma triggered by driver mutations, increased H3K9 methylation induced by SETDB1 displays an oncogenic effect in an established tumor. What’s more, the loss of H3K27 trimethylation due to *DOT1L* gene deletion also contributes to UVR-triggered melanomagenesis. Therefore, these reports highly indicate the bi-modal role of histone methylation during the whole process of melanoma pathogenesis, namely, being suppressive for carcinogenesis whereas facilitative for progression. Taken together, histone modification coordinates various hallmark characteristics of cancer including cell metabolism, genome instability, and immune evasion in melanoma, and histone methylation-targeted therapy should be based on the progression stage.

In addition to DNA methylation and histone modification, non-coding RNA and m^6^A RNA methylation are additional pivotal paradigms of epigenetic modification. The expression profiles of microRNAs, lncRNAs, and circRNAs in melanoma have been extensively investigated and are found to be related to multiple cancer characteristics like metastasis, cellular metabolism, migration and invasion, and antitumor immunity.^172–180^ Investigations of miRNAs in melanoma initially focused on their roles in hallmark characteristics of cancer biology.^181–184^ Then, more and more attentions were paid to their roles in the tumor microenvironment including angiogenesis, metastatic niche formation, and T cell dysfunction.^182,185,186^ It should be noted that the biological functions of some non-coding RNAs are of high specificity in melanocytic lineage. For example, Gilot et al. has demonstrated that the sequestration of miR-16 by the mRNA of melanocyte specifically-expressed TYRP1 can promote tumor growth by relieving the suppression of downstream tumor-promoting factors like RAB17, highlighting miRNA displacement as a promising therapeutic approach.^187^ The crosstalk between melanocytic lineage-specific factor and non-coding RNA is further extended by the investigation conducted by Leucci et al., which demonstrates that recently annotated lncRNA SAMMSON is co-amplified with MITF and plays an oncogenic role. As a transcriptional target of SOX10, the expression of SAMMSON is detectable in more than 90% of melanomas. The knockdown of SAMMSON disrupts mitochondrial functions by targeting downstream p32 and can thereby increase the efficacy of MAPK inhibition-targeted therapy^69^ (Fig. 5). These reports emphasize that the function of non-coding RNAs can be exerted in a cancer-type-specific manner. The current progress of lncRNAs in melanoma pathogenesis is summarized in Table 2. Apart from non-coding RNA, m^6^A RNA methylation is another crucial chemical modification discovered in mRNA and non-coding RNA in eukaryotic cells,^188,189^ the process of which is dynamically regulated by a series of “writers”, “readers”, and “erasers”.^190^ In melanoma, the expression of m^6^A demethylase FTO is significantly upregulated to contribute to not only tumorigenicity but also increased response to anti-PD-1 blockade by orchestrating the expressions of PD-1, CXCR4, and SOX10.^191^ Other reports also provide evidence that m^6^A demethylase alkylation repair homolog 5 (ALKBH5) and methyltransferases METTL3/14 regulate the response to anti-PD-1 blockade by re-shaping tumor microenvironment through the regulation of metabolism and chemokine secretion.^192,193^ The role of m^6^A methylation in tumor-infiltrating macrophages and related impact on tumor progression have also been elucidated in melanoma. Loss of METTL3 in myeloid cells can prominently impair YTH N6-methyladenosine RNA binding protein-1 (YTHDF1)-mediated translation of sprouty related EVH1 domain-containing 2 (SPRED2), which promotes the activation of nuclear factor kappa B (NF-kB) and STAT3 through ERK pathway, leading to increased tumor growth and metastasis. This regulatory mechanism is also implicated in the regulation of the response to anti-PD-1 immunotherapy^194^ (Fig. 5). Given the pivotal role of non-coding RNA and m^6^A methylation in melanoma pathogenesis elucidated by previous reports, further investigations in this area can bring more insights leading to innovative advances for melanoma therapy.

**Table 2:** Current progress of lncRNAs in melanoma pathogenesis

LncRNA	Expression status	Role in melanoma pathogenesis	Binding partner/target	Effect on downstream signal pathways	Reference (PMID number)
BASP1-AS1	Upregulated	Promoter	YBX1	Notch activation	34533860
SAMMSON	Upregulated	Promoter	P32	Promote mitochondrial function	27008969
NCK1-AS1	Upregulated	Promoter	miR-526b-5p	ADAM15 upregulation	34247598
FUT8-AS1	Downregulated	Suppressor	NF90	NRAS/MAPK	34094894
LINC00470	Upregulated	Promoter	N.A.	Promote APE1 expression	33875645
LINC01291	Upregulated	Promoter	miR-625-5p	Promote IGF-1R expression	33674778
TINCR	Downregulated	Suppressor	ATF4 mRNA	Prevent ATF4 translation and expression	33586907
MIR205HG	Upregulated	Promoter	miR-299-3p	Promote VEGF-A expression	33535182
LINC00518	Upregulated	Promoter	N.A.	Affect multiple events like EMT and hypoxia-like response	33371395
NEAT1	Upregulated	Promoter	miR-200b-3p	SMAD2 activation	33202380
LHFPL3-AS1	Upregulated	Promoter	miR-181a-5p	Promote BCL2 expression and stem cells survival	33149126
TTN-AS1	Upregulated	Promoter	TTN	Promote TTN expression	32820147
LHFPL3-AS1	Upregulated	Promoter	miR-580-3p	STAT3 activation	32753471
LINC00520	Upregulated	Promoter	miR-125b-5p	Promote EIF5A2 expression	32466797
SRA	Upregulated	Promoter	N.A.	P38 activation and EMT	31945347
MEG3	Downregulated	Suppressor	miR-21	E-cadherin upregulation	31938020
LINC-PINT	Downregulated	Suppressor	EZH2	Increased H3K27 trimethylation and epigenetic rewiring	31921860
DIRC3	Downregulated	Suppressor	N.A.	IGFBP5 upregulation	31881017
LINC00518	Upregulated	Promoter	miR-204-5p	AP1S2 upregulation	31712557
FOXD3-AS1	Upregulated	Promoter	N.A.	MAP3K2 activation	31541886
ZNNT1	N.A.	Suppressor	N.A.	Autophagy activation	31462126
Linc00961	Downregulated	Suppressor	miR‑367	PTEN upregulation	31364744
LNMAT1	Upregulated	Promoter	EZH2	Downregulation of CADM1	31334110
SLNCR1	Upregulated	Promoter	AR and EGR1	Downregulation of P21	31116991
CPS1-IT1	Downregulated	Suppressor	BRG1	Downregulation of CYR61	31111478
OIP5-AS1	Upregulated	Promoter	miR-217	Promote GLS expression and glutamine catabolism	30779126
CASC15	Upregulated	Promoter	EZH2	PDCD4 downregulation	30013768
LncRNA-ATB	Upregulated	Promoter	miR-590-5p	Promote YAP1 expression	29956757
KCNQ1OT1	Upregulated	Promoter	miR-153	Suppress MET expression	29667930
CASC2	Downregulated	Suppressor	miR-18a-5p	RUNX1 downregulation	29422114
HOXD-AS1	Upregulated	Promoter	EZH2	RUNX3 upregulation	29312805
FALEC	Upregulated	Promoter	EZH2	p21 downregulation	29196104
BANCR	Upregulated	Promoter	miR‑204	Notch2 upregulation	29075789
CCAT1	Upregulated	Promoter	miR-33a	N.A.	28409554
PVT1	Upregulated	Promoter	miR-26b	N.A.	28409552
RHPN1-AS1	Upregulated	Promoter	N.A.	N.A.	28124977
NKILA	Downregulated	Suppressor	N.A.	Suppression of NF-ĸB	28123845
ANRIL	Upregulated	Promoter	PRC1	Repress the expression of CDKN2A	20541999
SLNCR1	Upregulated	Promoter	N.A.	MMP9 upregulation	27210747
SAMMSON	Upregulated	Promoter	p32	Increased mitochondrial function	27008969
CDR1as	Downregulated	Suppressor	IGF2BP3	N.A.	31935372
GAS5	Downregulated	Suppressor	E2F4	Repress E2F4 expression	32308561
HEIH	Upregulated	Promoter	EZH2	Inhibition of miR-200 cluster	28487474
TSLNC8	Upregulated	Promoter	PP1α	MAPK reactivation	33389075
KCNQ1OT1	Upregulated	Promoter	miR-153	Repress MET expression	29667930
LINC00459	Downregulated	Suppressor	miR-218	DKK3 activation	31844121
LINC01158	Upregulated	Promoter	miR-650	MGMT upregulation	33816296
MALAT1	Upregulated	Promoter	miR-34a	c-Myc, MET	31101802
ZEB1-AS1	Upregulated	Promoter	miR-1224-5p	N.A.	30651872

In general, compared to genetic variations, epigenetic modifications are more accessible and easily reversible, providing more options to develop promising drugs like HDAC inhibitors and EZH2 inhibitors. However, the non-specific characteristic of epigenetic modification determines that intervening epigenetics might not be as precise as that of targeted therapy for cancer treatment, and can lead to more side effects.^195^ Additional investigations are needed to improve the specificity of epigenetic modulation-based cancer therapy, as well as lowering the toxicity.

### Signal pathways implicated in metabolic reprogramming

Metabolic reprogramming is a hallmark characteristic of cancer. The metabolism of melanoma cells is of rather high plasticity and builds a bridge that connects oncogenic factors to energetic supplement. Multiple paradigms of cellular metabolism like glycolysis, lipid metabolism, amino acid metabolism, nucleotide metabolism, oxidative phosphorylation and autophagy participate in not only the malignant behavior of melanoma cells, but also the re-establishment of the tumor microenvironment and the regulation of tumor-infiltrating immune cells.^196^ Some key metabolic enzymes have been considered as promising intervening targets to restrain melanoma progression, as well as to synergize with targeted therapy and immunotherapy.^197,198^

**Fig. 6:**
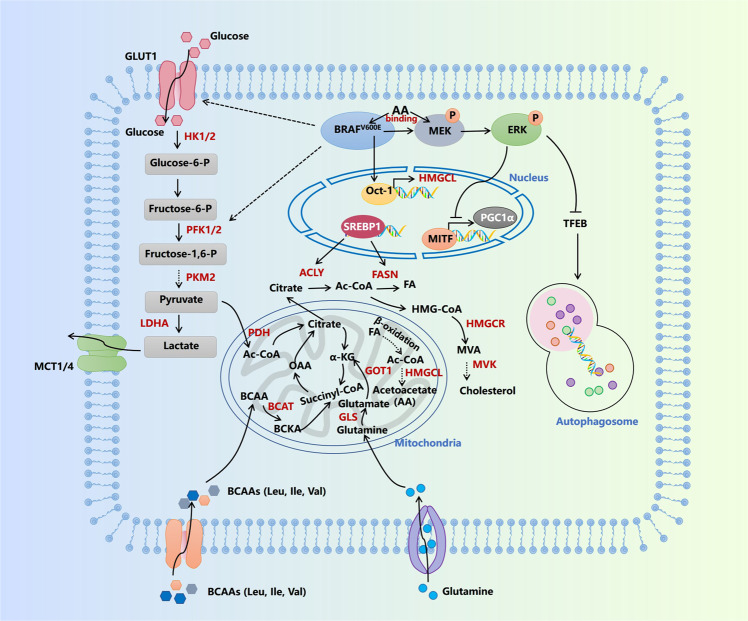
Signal pathways of metabolic reprogramming in melanoma. AA acetoacetate, ACLY ATP-citrate lyase, BCAT branched-chain amino acid transaminase, BCKA branched-chain keto acids, FA fatty acid, FASN fatty acid synthase, GLS glutaminase, GLUT1 glucose transporter type 1, GOT1 glutamicoxaloacetic transaminase 1, HK1/2 hexokinase 1/2, HMGCL 3-hydroxy-3-methylglutaryl-CoA lyase, HMGCR 3-hydroxy-3-methylglutaryl-CoA reductase, LDHA lactate dehydrogenase A, MCT1/4 monocarboxylate transporter, MVA mevalonate, MVK mevalonate kinase, OAA oxaloacetate, PDH pyruvate dehydrogenase, PFK1/2 phosphofructokinase 1/2, PKM2 pyruvate kinase M, SREBP-1 sterol regulatory element-binding transcription factor 1, TFEB transcription factor EB

Aerobic glycolysis, also termed as the Warburg effect, is the most common metabolic characteristic in many cancers, with no exception in melanoma. Metabolite profiling reveals that the most frequently-occurred *BRAF* mutation endows melanoma cells with enhanced glycolytic capacity, which can be attributed to the upregulation of a network of transcriptional factors, glucose transporters, and kinases controlling glycolysis including HIF1α, MYC, Glut 1, Glut 3, and Hexokinase 2 (HK2).^161,199^ Moreover, BRAF negatively regulates MITF–PGC1α axis to suppress oxidative phosphorylation, which indicates that BRAF-driven glycolysis also partially results from the compensatory activation after the inhibition of mitochondrial function.^78,200^ Supplementary to transcriptional regulation, the downstream kinase of hyper-activated MAPK pathway ribosomal protein S6 kinase (RSK) can directly phosphorylate and activate 6-phosphofructo-2-kinase/fructose-2,6-bisphosphatase 2 (PFKFB2), an enzyme that catalyzes the synthesis of fructose-2,6-bisphosphate during glycolysis, to facilitate *BRAF*-driven glycolytic metabolism^201^ (Fig. 6). Enhanced glycolysis provides enriched metabolic intermediate as building bricks to promote the synthesis of macromolecules like proteins, nucleic acid, and lipid acid.^202^ Moreover, the accumulation of intracellular lactate, the end product of glycolysis, leads to extracellular acidification via monocarboxylate transporter 4 (MCT-4), which impedes the function of tumor-infiltrating CD8^+^T lymphocytes.^203^ Therefore, the inhibition of glycolysis and lactate production can increase the treatment efficacy of anti-PD-1 immunotherapy via the reactivation of antitumor immunity.^204,205^ In addition to enhanced glycolysis, there are some data supporting a specific role of *BRAF* mutation on oncogenic metabolism. By using a shRNA library covering the known metabolism-related enzymes and protein factors in the human genome, 3-hydroxy-3-methylglutaryl-CoA (HMGCL), the rate-limiting enzyme implicated in ketogenesis, is identified as a “synthetic lethal” partner of *BRAF* mutation in melanoma cells.^206^ To be specific, mutant *BRAF* can transcriptionally upregulate HMGCL via Oct-1, and the end product of ketogenesis acetoacetate (AA) catalyzed by HMGCL further promotes the interaction between BRAF^V600E^ and MEK1 to amplify MAPK activation (Fig. 6). Therefore, *BRAF*-mutant melanomas are addicted to ketogenesis.

Mitochondrial oxidative phosphorylation is generally considered to be suppressed during tumor progression as a result of the Warburg effect. However, previous studies have provided compelling evidence that mitochondrial function is essential for maintaining tumor cell survival in melanoma and contributes greatly to melanoma growth. Vazquez et al. firstly found that a subset of melanomas harboring higher PGC1α expression has stronger capacity to defend against oxidative stress and rely on the mitochondrial function to survive and develop.^207^ This metabolic alteration is induced by MITF-mediated transcriptional upregulation, which is negatively regulated by BRAF.^200^ Upon the inhibition of the MAPK pathway by BRAF-targeted therapeutic agent, mitochondrial oxidative phosphorylation would be activated as a result of MITF–PGC1α axis activation, rendering the resistance to treatment.^158,208^ Moreover, lncRNA SAMMSON that co-expresses with MITF promotes melanoma growth by directly regulating mitochondrial master regulator p32, the inhibition of which significantly impedes the survival capacity of tumor cells.^209^ In contrast to the essential effect on tumor growth, PGC1α orchestrates a transcriptional axis that suppresses melanoma metastasis.^210^ Moreover, mitochondrial gatekeeper pyruvate dehydrogenase (PDH) is proved as a crucial mediator of BRAF-induced senescence. The suppression of PDH and relevant mitochondrial function is capable of abrogating BRAF-induced senescence, thereby licensing BRAF-driven melanoma development.^211^ Therefore, mitochondrial function switches from tumor suppressor to oncogenic factor during melanoma carcinogenesis and development. The function of mitochondria in melanoma pathogenesis seems paradoxical and is rather similar to that of histone methylation. Increased mitochondrial oxidative phosphorylation is essential for melanoma cell proliferation, whereas acts as an obstacle in melanomagenesis. Moreover, mitochondria master regulator PGC1α is also related to the phenotype switch between proliferative and invasive state, which is due to the discrepancy of downstream predominant activated signal pathways. Therefore, the intervention of PGC1α expression for melanoma therapy should take the clinical stage into consideration. Of note, a recent study using proteomics analysis demonstrates that the sensitivity to immunotherapy is highly related to higher oxidative phosphorylation and lipid metabolism in tumor cells,^212^ expanding the pathologic implication of mitochondrial function in melanoma.

The disorder of lipid metabolism is another hallmark metabolic characteristic of melanoma. Potentiation of lipogenesis and enhanced lipid uptake endow tumor cells with proliferative advantage by not only constituting the structure of multiple biological membranes but also providing an energy source.^213^ De novo fatty acid synthesis is regulated by a series of enzymes including ATP-citrate lyase (ACLY), acetyl-CoA carboxylase (ACC), fatty acid synthase (FASN), and acyl-CoA synthetase (ACS), whereas cholesterol biogenesis is orchestrated by acetyl-CoA acetyltransferase 2 (ACAT2), 3-hydroxy-3-methylglutaryl-CoA synthase (HMGCS), 3-hydroxy-3-methylglutaryl-CoA reductase (HMGCR) and mevalonate kinase (MVK). These two pathways are governed by sterol regulatory element-binding protein-1 (SREBP-1) and SREBP-2 respectively in a transcription-dependent manner^214^ (Fig. 6). The expressions of these lipid metabolism regulatory factors are generally increased in melanoma, and the inhibition of them can lead to prominent tumor regression,^158,215–222^ indicating the essential role of lipid biosynthesis for melanoma cell proliferation. In particular, integrative analysis with the use of positron emission tomography (PET), desorption electrospray ionization-mass spectrometry (DESI-MS), nonimaging MS and transcriptomic analyses in the zebrafish melanoma model provides direct evidence of increased lipid uptake and dysregulated glycerophospholipid metabolism.^215^ Targeted inhibition of fatty acid receptor CD36 can restrain the potential of metastasis-initiating cells and thereby impair tumor metastasis.^223^ What should be noted is that the lipogenic enzymes ACLY and SREBP-2 also have some lipogenesis-independent tumorigenic functions, with the engagement of mitochondrial oxidative phosphorylation and iron metabolism respectively.^158,216^ In addition to this, some metabolic intermediates in lipid metabolism like acetyl-CoA and palmitic acid are highly related to histone modification and non-histone modification,^158,224^ highlighting the close relationship between metabolism and protein modification. To be specific, palmitic acid can induce palmitoylation of cysteine residues of MC1R to trigger its activation and regulate downstream pigmentation and cell-cycle arrest under UVB radiation, which plays a protective role in preventing melanomagenesis.^224,225^ Moreover, increased acetyl-CoA produced by ACLY promotes histone acetylation and activates the transcription of the MITF–PGC1α axis to facilitate melanoma growth.^158^ More importantly, the upregulation of lipogenic enzymes like ACLY and SREBP-1 can also mediate the resistance to targeted therapy in melanoma,^158,226^ indicating that targeting lipogenesis is also a promising therapeutic approach to reinforce the treatment efficacy of MAPK inhibition.

Autophagy is a crucial metabolic process characterized by delivering intracellular proteins and organelles into autolysosomes for digesting and recycling, which physiologically provides intermediates as building bricks for macromolecules biogenesis, and sufficient ATP for cell survival and homeostasis.^227^ Liu et al. initially discovered that low expression of autophagy regulator autophagy-related 5 (ATG5) helps to overcome BRAF-induced senescence to promote the malignant transformation of early-stage melanoma.^228,229^ Besides, the deficiency of autophagy also facilitates tumor metastasis by stabilizing Twist family BHLH transcription factor 1 (TWIST1) protein through p62 accumulation.^230^ These reports indicate autophagy as a potential tumor suppressor in melanoma. In contrary, by employing a well-established melanoma mice model induced by both *BRAF* mutation and loss of *PTEN*, Xie et al. reported that ATG7 deficiency could restrain tumor progression by increasing oxidative stress and inducing cell senescence.^231^ This finding has been further supported by Rosenfeldt et al, while the tumorigenic role of ATG7 in vivo relies on the status of PTEN.^232^ Moreover, autophagy-driven ATP secretion also contributes to the invasive and migratory capacity of melanoma cells through the purinergic receptor P2RX7.^233^ The above-mentioned paradoxical roles of autophagy in melanoma have been clarified by our group based on the results of the fluctuation of autophagy level during melanoma progression. In particular, autophagy level is downregulated in the early stage but upregulated in metastatic stage, which is under the control of histone deacetylase sirtuin 6 (SIRT6) via epigenetic modulation of insulin-like growth factor (IGF)-AKT pathway. More importantly, autophagy indeed plays a bi-modal role in melanoma growth at different clinical stages, namely, tumor suppressor at early stage but tumor promoter at advanced stage.^234^ Other regulators including BRAF, miR-23a, transcription factor EB (TFEB), unfolded protein response (UPR) machineries, and receptor-interacting serine/threonine kinase 1 (RIPK1) are also responsible for increased autophagy level under either normal or stressful conditions in melanoma.^181,235–237^ Intriguingly, the activation of autophagy has also been observed in melanomas resistant to BRAF-targeted agents, which significantly hinders the treatment effect and renders therapeutic resistance.^238^ Therefore, targeting autophagy should be emphasized as a potential therapeutic approach for not only restraining tumor progression, but also increasing the efficacy of targeted therapy in melanoma.^239,240^

The dysregulation of amino acid metabolism, especially that of serine, glutamine, and branched-chain amino acid, is also greatly involved in melanoma pathogenesis. The rate-limiting enzyme of de novo serine synthesis is 3-phosphoglycerate dehydrogenase (PHGDH), of which the copy number is reported to be significantly increased in melanoma to ensure tumor cell survival and proliferation under low physiological serine concentrations.^241,242^ Supplement of dietary serine or genetic overexpression of PHGDH can efficiently foster melanoma progression via the enhancement of intracellular serine level.^242^ More intriguingly, PHGDH is also decisive in mediating melanoma metastasis to the brain. With the use of proteomics, metabolomics, and multiple brain metastasis models, it has been revealed that the metastatic lesions of melanoma is sensitive to the limitation of serine synthesis in nutrient-limited environment, making PHGDH an attractive target to control melanoma distant metastasis.^243^ Recent study regarding targeted therapy forwardly shows that PHGDH upregulation also renders the resistance to MEK inhibitor in melanoma harboring *NRAS* mutation. Targeting PHGDH can re-sensitize resistant tumors to MAPK inhibition via the reduction of glutathione and the increment of oxidative stress.^244,245^ Therefore, PHGDH inhibition is a lethal partner with MAPK inhibitor for melanoma therapy. Apart from serine metabolism, melanoma cells are also addicted to glutamine for fueling tumor progression. Glutamine metabolism is orchestrated by a series of machineries including glutamine importer, glutaminases 2 (GLS2) and glutamicoxaloacetic transaminase 1 (GOT1) to promote glutamine uptake and subsequent formation of glutamate and α-ketoglutarate (α-KG) (Fig. 6). Targeting either glutamine transport or glutaminase can obtain prominent regression of tumor.^246,247^ Moreover, glutamine acts as a compensated source supporting melanoma cell survival in case of the inhibition of lactate dehydrogenase A (LDHA)-mediated Warburg effect through activating transcription factor 4 (ATF4)-mediated upregulation of glutamine transporter,^248^ or after the suppression of mitochondrial oxidative phosphorylation by PGC1α knockdown.^249^ In the tumor microenvironment, decreased α-KG level caused by regional loss of glutamine leads to histone hyper-methylation and thereafter cell de-differentiation to render melanoma cells more resistance to targeted therapy.^250^ Nevertheless, melanoma cells with the acquired resistance to BRAF inhibitor are highly dependent on glutamine for cell proliferation,^251^ indicating that glutamine metabolism might play contrary roles in different phases of treatment resistance. Although the blockade of intra-tumoral glutamine metabolism results in impeded tumor cell proliferation, dietary glutamine supplementation could suppress melanoma growth in vivo via α-KG-mediated epigenetic rewiring,^252^ indicating a proposed model that excessive glutamine uptake might otherwise abrogate the essential effect of glutamine on supporting tumor cell survival. In addition to serine and glutamine, branched-chain amino acids (BCAAs) comprising of leucine (Leu), isoleucine (Ile), and valine (Val), as well as their metabolism, are implicated in cancer pathogenesis. After the intake by tumor cells, branched-chain amino acid transaminase 1 and 2 (BCAT1/2) transfers nitrogen of BCAAs to α-ketoglutarate (αKG) to produce glutamine and branched-chain keto acid (BCKA), which is then metabolized by branched-chain alpha-keto acid dehydrogenase complex (BCKDH) and some other enzymes to produce tricarboxylic acid (TCA) cycle intermediates acetyl-CoA and/or succinyl-CoA, thus fueling tumor cell proliferation and providing some building bricks to biogenesis^253^ (Fig. 6). As is similar to other types of cancers, the expression of BCAT1 is significantly upregulated in melanoma. Genetic knockdown of BCAT1 expression impairs the proliferative capacity of melanoma cells via the suppression of mitochondrial oxidative phosphorylation.^254^ Moreover, genome-wide CRISPR/Cas9 knockout screening assay has identified that dihydrolipoamide branched-chain transacylase (DBT), a subunit of BCKDH, is implicated in the regulation of cell apoptosis induced by BRAF^V600E^ overexpression in melanocytes. The inhibition of DBT contributes to BCAAs accumulation and attenuates oncogene-induced apoptosis of melanocytes, suggesting that DBT is a gatekeeper mediating mutant BRAF-driven malignant transformation from melanocytes to melanoma.^255^ What’s more important, melanoma cells harboring *BRAF* mutation and hyper-activation of the MAPK pathway are highly dependent on leucine for survival. The inhibition of autophagy mimicking activated RAS-MEK signaling renders tumor cells to leucine deprivation, so that dietary leucine deprivation and autophagy inhibition could synergistically suppress melanoma growth.^256^ Therefore, BCAAs metabolism is a promising therapeutic target for not only preventing the malignant transformation of melanocytes but also suppressing melanoma progression.

In aggregate, melanoma cells are of high metabolic flexibility and have a complicated metabolic network with the involvement of multiple paradigms including glycolysis, mitochondrial oxidative phosphorylation, lipid metabolism, autophagy, and amino acid metabolism. These metabolic characteristics endow tumor cell with growth advantages by supplying not only sufficient energy, but also abundant metabolic intermediates for the synthesis of various macromolecules, which are essential for rapid cell proliferation. Of note, there are some metabolic alterations specific to oncogenic mutations or melanocytic lineage, including mutated *BRAF*-driven ketogenesis and SOX10-mediated SAMMSON upregulation and mitochondrial oxidative phosphorylation, which might provide an accessible target for a specific subgroup of patients with melanoma. However, the frequent occurrence of compensated activation of alternative pathways prominently hinders the efficacy of targeting one single metabolic paradigm.^249,257^ Combinatorial suppression of multiple metabolic pathways might be more efficient in controlling melanoma growth, which needs more investigations in the future.

### Key signal pathways in tumor metastasis

Tumor metastasis is the most important cause of the unoptimistic prognosis of melanoma patients.^258^ Generally, the occurrence of metastasis mainly includes the following key steps, namely, invasion, intravasation, circulation, extravasation and colonization at secondary tumor sites,^259^ which are orchestrated by a series of distinct biological principles.^260^ Herein, we emphasize epithelial–mesenchymal transition (EMT), melanoma cell adhesion, and exosomes to delineate the signal pathways landscape and hope to generate mechanistic insights of melanoma metastasis.

EMT refers to a cellular program generally characterized by the downregulation of multiple epithelial markers like E-cadherin, laminin, cytokeratin, and the upregulation of mesenchymal markers like N-cadherin, vimentin, and α-SMA. EMT is physiologically fundamental in embryogenesis, fibrosis, and wound healing.^260^ In cancer, the occurrence of EMT is accompanied by morphologic switch from an epithelioid toward a mesenchymal/spindle cell shape, endowing tumor cells with enhanced invasive and migratory capacity. Previously, a high-throughput gene-expression profile has identified EMT as a major determinant of melanoma metastasis.^261^ Subsequent mounting evidence has revealed the pivotal role of EMT in facilitating melanoma metastasis and the upstream regulatory network.^262^ To be specific, under the control of some canonical oncogenic pathways like BRAF/MEK, AKT/mTOR, Wnt/β-catenin and TGF-β, the switch of the expressions of EMT-inducing transcriptional factors (EMT-TF) including MITF, SOX2, Snail, Slug, TWIST1, zinc finger E-box binding homeobox 1/2 (ZEB1/2) and NF-κB governs the irreversible process of EMT.^262^ In response to the activation of MEK/ERK in melanomas harboring *BRAF* or *NRAS* mutation, molecular network of EMT-TF is profoundly re-organized to be in favor of TWIST1 and ZEB1, whereas the expressions of Snail2 and ZEB2 are deficient. This alteration is highly attributed to ERK activation and the resultant induction of AP-1 family member FRA1, cooperating with *BRAF* mutation to drive the gain of invasive ability and tumor metastasis.^263^ In addition to BRAF/MEK cascade, AKT/mTOR signal is greatly engaged in melanoma metastasis as well. The activation of AKT and mTOR downstream effector 4E-BP1 is in strongly positive correlation with the aggressiveness of melanoma, and the poor prognosis of patients in two independent cohorts.^264–267^ Loss of PTEN is the main reason for hyper-activation of AKT pathway, and several integrated molecular and clinical analysis all point out the close relationship between AKT activation and the occurrence of metastasis to specific regions like brain, lymph nodes, and lungs, accompanied with the upregulation of β-catenin and the downregulation of E-cadherin.^110,268–270^ Moreover, overexpression of AKT dramatically induces invasive phenotype of melanoma, not only promoting the conversion from radial to vertical growth, but also contributing to metastasis to distant organs in established melanoma transgenic mice model,^42,271–273^ with EMT responsible for this phenotypic switch.^274–276^ Aside from MAPK and AKT pathway, Wnt/β-catenin has also been closely related to EMT and tumor metastasis in melanoma. β-catenin is a central mediator of metastasis in established transgenic mice model harboring both *BRAF* gain-of-function mutation and *PTEN* loss-of-function mutation via simultaneous activation of MAPK and AKT pathways.^110^ Clinically, the activation of non-canonical Wnt5a signaling is positively related to the clinical stage and survival of patients, and strong cytoplasmic Wnt5a staining is an independent risk factor for reduced metastasis-free and overall survival in multivariate analysis.^277,278^ Dissanayake et al. has demonstrated that Wnt5a suppresses PKC signaling to initiate EMT by increasing the expressions of Snail and vimentin, as well as the downregulation of E-cadherin.^279^ Moreover, Sinnberg et al. has proved that β-catenin is frequently expressed at the invasive front of melanoma, and Wnt/β-catenin promotes neural crest migration of melanoma cells and induces an invasive phenotype.^111^ Therefore, targeting Wnt/β-catenin could be exploited as a promising approach to impede melanoma metastasis by attenuating EMT. Of note, TGF-β is another vital player in melanoma metastasis. In tumor microenvironment, mesenchymal stem cell-derived TGF-β promotes melanoma cell EMT in paracrine/autocrine-dependent manner.^280^ What should be noted is that TGF-β-mediated transcriptional profile also facilitates ameboid phenotype independent of the EMT process to promote melanoma cell dissemination.^281^ Downstream the above-mentioned oncogenic pathways, EMT-TFs are indeed master regulator of melanoma phenotype switching in a transcriptional regulation-dependent manner. Wels et al. has discovered the specifically regulatory effect of Slug, rather than Snail or Twist, on the upregulation of ZEB1 and downregulation of E-cadherin, thus resulting in decreased adhesion to human keratinocytes, and enhanced migration of melanoma cells.^282^ Another investigation with the employment of in vivo fate mapping technology demonstrates that melanoma cells undergo a conversion in state where ZEB2 expression is replaced by ZEB1 expression associated with gain of an invasive phenotype, suggesting that reversible switching of the ZEB2/ZEB1 ratio could enhance melanoma metastatic dissemination.^283^ Meanwhile, loss of ZEB2 expression results in prominent downregulation of MITF and relevant differentiated phenotype, concomitant with an upregulation of ZEB1 and mesenchymal characteristic of melanoma cells. The crosstalk among these three crucial EMT-TFs orchestrates the transcriptional program of phenotypic plasticity.^284^ As the melanocytic lineage-specific transcriptional factor, MITF exerts an integrated effect on suppressing focal adhesion and N-cadherin expression, thus ameliorating the local detachment and dissemination of tumor cell.^285^ What’s more, the stabilization and accumulation of TWIST1 due to autophagy deficiency and increased SQSTM1-TWIST1 interaction is also an important trigger of EMT in melanoma.^230^ Taken together, the regulatory network of EMT-TFs is a pivotal contributor and potent therapeutic target of melanoma metastasis.

The adhesiveness and intercellular communication within the tumor microenvironment are also decisive for tumor cell dissemination, with cell-adhesion molecules (CAM) governing this process. CAM includes a large family of proteins located on cell surface like integrin, cadherin, IgSF, connexin and mucin that regulate attractive or repulsive forces to the extracellular matrix (ECM), stroma and other cancer cells, thus affecting the invasive and migratory capacity.^286^ Integrins are heterodimeric proteins on the cell membrane that adhere to the ECM and can also sense and transduce extrinsic signalings.^287^ Besides, at least 18 α- and 8 β-subunits constitute the presently-known 24 heterodimers of the superfamily integrins.^288^ Initial reports have demonstrated the dysregulation of multiple integrins in regulating melanoma cell survival, tumor growth, tumor metastasis, and the association with clinical characteristics.^289–294^ To be specific, previous reports from Herlyn’s group have reported that increased expression of integrin ɑvβ3 is associated with growth and the conversion from radial growth phase (RGP) to vertical growth phase (VGP) in melanoma.^295,296^ In addition, integrins like ɑ2β1, ɑ5β1, and ɑvβ3 can directly stimulate the expression and function of matrix metallopeptidases (MMPs), which facilitates the degradation of collagen and fibronectin, so as to contribute to tumor cell invasion and progression.^297–299^ Of note, targeted blockade of either integrins β1 or ɑvβ3 leads to prominent suppression of tumor cell adhesion and migration.^300^ What’s more, integrins also play important role in promoting angiogenesis, which is implicated in supplying sufficient nutrient and orienting the spread of tumor cells to distant organs. In particular, fibroblast growth factor 2 (FGF-2) and vascular endothelial growth factor A (VEGF-A) promotes angiogenesis via the regulation of integrin ɑvβ3 and ɑvβ5 respectively, which is supported by the evidence that pharmacological blockade of ɑvβ3 and ɑvβ5 ameliorates increased angiogenesis induced by FGF-2 and VEGF-A.^301^ In addition to these, integrin-based signaling transduction and associated intermediaries have recently attracted more attention due to their impact on melanoma pathogenesis. For example, increased expression of integrin-linked kinase (ILK) is highly associated with progression of melanoma and the poor prognosis of patients.^302^ Genetic knockdown of ILK expression results in significant impairment of melanoma cell migration and the formation of anchorage-independent colonies in soft agar as well, indicating the indispensable role of ILK in melanoma development.^303^ In addition, the upregulation of ILK also contributes to melanoma angiogenesis via the enhancement of NF-κB and IL-6 signaling.^304^ Recently, Gil et al. has demonstrated that the deficiency of ILK regulates the endosomal recycling of N-cadherin and reduces membrane N-cadherin expression to ameliorate melanoma metastasis.^305^ Huang et al. further pointed out that distinct integrins on melanoma cell specifically direct circulating melanoma cells to different organs and the establishment of metastases at specific organ sites.^306^ Besides, exosomes derived from tumor cell that contained different integrins contribute to the formation of pre-metastatic niche in targeted organs and promoted organ-specific metastases.^306^ Therefore, the pharmacological intervention of integrins has been broadly investigated in preclinical and clinical trials.^307–310^ What should also be mentioned regarding CAM is cadherin that refers to a family of calcium-dependent cell-adhesion proteins. The “cadherin switching” during tumor progression is generally characterized by the loss of E-cadherin and the increase of N-cadherin,^311^ which highly contributes to increased interactions between melanoma cells and dermal fibroblasts/vascular endothelial cells, and the impaired junction to keratinocytes. The E-cadherin/N-cadherin switch is highly associated with low or absent PTEN expression and disease progression in melanoma.^312^ Of note, N-cadherin-regulated cell-adhesion results in the potentiation of AKT-β-catenin signaling to antagonize the expressions of pro-apoptotic factors, exerting pivotal effect on tumor cell survival in addition to migratory ability.^313^ Aside from these, two reports have demonstrated that P-cadherin counteracts the invasion and migration of melanomas via the increase of cell-cell interaction, suggesting that more members of cadherins might play a role in melanoma pathogenesis.^314,315^

Exosomes are extracellular vesicles transporting proteins, nuclear acids, and metabolites that can mediate intercellular communication in the tumor microenvironment.^316^ Due to the resistance to proteolytic and nuclease activity, exosomes are relatively stable and cargos in them are protected from various stress and degradation.^317^ The capacity of exosomes to carry nuclear acids and proteins endows them with the function to regulate the metastatic ability of tumor cells in the primary region and remote organs or tissues that provide the soil the formation of metastases.^318^ Initially, Hood et al. discovered that exosomes released by melanoma cells define microanatomic responses in sentinel lymph nodes that licenses metastasis of melanoma cells. To be specific, homing of melanoma cells-derived exosomes to sentinel lymph nodes exerts integrated effects on melanoma cell recruitment, extracellular matrix deposition, and vascular proliferation in the lymph nodes, thus helping microanatomic niche preparation to facilitate tumor cell lymphatic metastasis.^319^ Later, it was unveiled that exosomes from highly metastatic melanoma could educate bone marrow progenitor cells toward a pro-vasculogenic phenotype and trigger vascular leakiness at pre-metastatic sites through the receptor tyrosine kinase MET. The formation and trafficking of exosomes are under the control of RAB27A, the knockdown of which significantly diminishes tumor metastasis.^320,321^ Given the great implication of exosomes in regulating tumor biology, the profiles of mRNA, miRNA and protein in melanoma cells-derived exosomes have been systemically analyzed, displaying specific signature related to metastatic potential.^322,323^ Of note, exosomes from melanoma cells can induce the phenotype switching of melanocytes, endowing them with increased invasive and metastatic capacity. For example, let-7i transferred by melanoma cell exosomes can induce epithelial–mesenchymal transition in primary melanocytes via the activation of MAPK signaling.^324^ In addition, exosomal miR-106b-5p derived from melanoma cell contributes to the EMT process of melanocytes by targeting EphA4 to activate the ERK pathway.^325^ Apart from these, exosomes also play a role in regulating angiogenesis and immune cell function in tumor microenvironment and preparation of a hospitable metastatic niche in distant organs to facilitate metastasis. For example, exosomes secreted by metastatic melanoma cells can instigate a pro-inflammatory gene signature in both lung fibroblasts and brain astrocytes, which promotes the formation of an inflammatory metastatic niche, suggesting that the reprogramming of stromal cells by tumor cell exosomes is a general mechanism in distant organs.^326^ Moreover, a series of reports have demonstrated that melanoma cell exosomes regulate the function of tumor-infiltrating immune cells to shape tumor microenvironment toward a pro-tumorigenic state. Gerloff et al. has reported that melanoma-derived exosomal miR-125b-5p induces a phenotype switch of tumor-associated macrophages toward a tumor-promoting state by targeting lysosomal acid lipase A.^327^ In addition, pre-metastatic tumors are capable of producing exosomes to potentiate immune surveillance by patrolling monocytes at the metastatic niche, indicating that exosomes from poorly metastatic melanoma cells can also potently inhibit metastasis to distant organ.^328^ In addition, some recent studies also demonstrate the involvement of tumor cell exosomes in regulating angiogenesis, metastatic niche formation, and mesenchymal stem cell oncogenic reprogramming.^329–331^ Therefore, exosomes exert a facilitative role in tumor metastasis via the regulation of multiple downstream biological activities.

The formation of metastasis is an integrative process with rather a complexity and mainly accounts for the mortality of melanoma patients. Extensive investigations have been conducted to elucidate the underlying mechanisms, which are far more than EMT, cell-adhesion alteration, and exosomes that we mentioned above. Metabolic rewiring, pre-metastatic niche formation, and the existence of dormancy have also been documented as critical characteristics of melanoma metastasis,^332^ which should also be taken into consideration for melanoma therapy.

### Signal pathways regulating oncogenic inflammation and angiogenesis

The inflammatory signal pathway is highly related to tumor carcinogenesis and progression, with no exception in melanoma.^333^ Inflammatory factors including tumor necrosis factor (TNFα), IFN-γ, interleukins, and related regulatory signalings such as Janus kinase (JAK)-STAT, NF-κB, and inflammasome have attracted more and more attention in the investigation of melanoma biology and tumor microenvironment.

A previous study using in situ hybridization assay firstly verified the existence of TNFα in melanoma cells in the tumor microenvironment,^334^ and the expression status of TNFα is related to driver mutation of the oncogene.^335^ In contrast to the fact that high-dose exogenous TNFα can induce apoptosis of melanoma which has been employed in various clinical trials and cancer therapy,^336,337^ TNFα derived from tumor cell or tumor microenvironment exerted a prominent regulatory role in tumor cell survival, proliferation, invasion, metastasis, and immune escape. Melanoma stimulated with recombinant TNFα displays downregulation of oncogenic factor c-myc, which thereby delays cell proliferation, indicating that TNFα antagonizes the outgrowth of tumor.^338^ However, tumor cells-secreted TNFα promotes downstream activation of RIPK1-NF-κB cascade in an autocrine manner to enable tumor cell survival, which is based on the observation that the deficiency of TNFαR1 or neutralizing TNFα in culture supernatant abrogates the activation of NF-κB signaling and restrains the proliferation of melanoma cell,^339^ highlighting TNFα as an oncogenic inflammatory factor. This conclusion is further supported by the results that in response to targeted inhibition of the MAPK pathway, TNFα enabled tumor cell survival by inducing c-FLIP upregulation and NF-κB activation.^340,341^ Therefore, the blockade of TNFα could be a promising synergized therapeutic approach with targeted therapy. Besides, Zhu et al. has provided evidence that TNFα stimulates the migratory potential of melanoma cells via the upregulation of fibronectin and integrin expressions, counteracting the suppressive effect of α-MSH.^342,343^ Consistent with this, TNFα promotes the expression of MMP2 and MMP9 to facilitate tumor cell migration.^344,345^ More importantly, it has been revealed that TNFα determines the phenotypic plasticity of melanoma cells by antagonizing MITF expression via downstream c-Jun. Dedifferentiated state of melanoma cells characterized by low MITF level has a higher inflammatory responsiveness and pathway activity. Clinically, the expression ratio of MITF and c-Jun could reflect the recruitment and infiltration of myeloid cells in tumor microenvironment, thus dictating the sensitivity to myeloid cells-directed immunotherapy.^346^ Some recent studies have also emphasized the role of TNFα in anti-tumor immunity and immunotherapy. Bertrand et al. demonstrated that the blockade of TNFα or TNFαR1 could enhance CD8^+^T cells-dependent antitumor immunity in established melanoma.^347^ Moreover, genome-wide screening uncovered that the ablation of TRAF2 could lower the TNFα cytotoxicity threshold in tumors by redirecting TNFα signaling to favor RIPK1-dependent apoptosis, thus increasing the susceptibility of tumors to immunotherapy.^348^ Further investigation also revealed that TNFα blockade could overcome the resistance to anti-PD-1 in melanoma via the prevention of cell death of tumor-infiltrating lymphocytes.^347^ In aggregate, targeting TNFα might be of high translational potential to synergize with anti-PD-1 antibody in treating melanoma. Of note, previous studies have shown the bifurcated functions of TNFα on melanoma pathogenesis, which is possibly determined by the source and the dosage of TNFα for the stimulation of melanoma cell. While high-dose exogenous TNFα mainly induces tumor cell apoptosis, endogenous low-concentration TNFα derived from tumor cell or tumor microenvironment contrarily plays an oncogenic role.

IFN-γ is enriched in the tumor microenvironment due to the infiltration of cytotoxic CD8^+^T lymphocytes. Physiologically, IFN-γ could activate JAK-STAT signaling to promote the expression of genes to defend against pathogen and infection. Recent studies mainly concentrate on the role of IFN-γ signaling in the regulation of tumor immune evasion and the implication in immunotherapy for melanoma. To be specific, IFN-γ could induce the expressions of multiple immune checkpoints including cytotoxic T lymphocyte antigen-4 (CTLA-4), PD-L1, and PD-L1 via JAK-STAT-dependent transcriptional cascade.^349–351^ The facilitation of PD-L1 by IFN-γ in melanoma cells is highly related to p53 expression.^350^ These data indicate that IFN-γ might modulate tumoral immune checkpoint to terminate the immune surveillance of tumor cell performed by lymphocytes, namely, immune evasion. Th1/IFNγ gene signature in the tumor microenvironment has been regarded as an independent biomarker to predict the prognosis of resectable high-risk melanoma patients.^352^ Moreover, the status of IFN-γ is associated with the response or resistance to immunotherapy. For instance, upregulated IFN-γ-related mRNA profile could predict better response to immunotherapy and better survival of patients.^353^ In line with this, Grasso et al. has also demonstrated that conserved IFN-γ transcriptome could drive the amplification of antitumor immune response and better treatment outcome of immune checkpoint blockade.^354^ Intriguingly, IFN-γ secreted by tumor-infiltrating lymphocytes after immunotherapy or radiotherapy could exert its direct effect on tumor cells via the downregulation of glutamate-cystine antiporter system Xc^-^ to trigger ferroptosis, a novel cell death modality characterized by excessive lipid oxidation, further supporting the facilitative role of IFN-γ in immunotherapy.^355,356^ However, there are two studies raising the notion that sustained activation of IFN signaling might be the cause of resistance to immune checkpoint blockades.^357,358^ The discrepancy could be related to the different phases and characteristics of intrinsic resistance and adaptive resistance in immunotherapy. Aside from IFN-γ, IFN-α is another crucial interferon with integrated functions of tumor control and immune regulation.^359–362^ In particular, IFN-α could stimulate host anti-tumor immunity by promoting the expression of major histocompatibility complexes (MHC) on tumor cell membrane, dendritic cell maturation, the cytotoxicity of natural killer (NK) cell, as well as the capacity of CD8^+^T cells for eradicating tumor cells.^361–363^ Although high-dose IFN-α has been approved for the treatment of resected melanoma as adjuvant therapy, the adverse effect is considerable and then is replaced by anti-PD-1 and anti-CTLA-4 immunotherapy.^364^ What should be noted is that IFN-α1b is reported to exert a better safety profile compared to IFN-α1a and is more tolerable for melanoma treatment. Prolonged usage of IFN-α1b in patients with unresectable metastatic melanoma has gained encouraging outcome.^365^

Interleukins and chemokines are two alternative pivotal types of inflammation-related factors in melanoma pathogenesis, especially in the regulation of immune cell function in the tumor microenvironment.^366^ Different interleukins exert distinct, even contrary effects on antitumor immunity. IL-2 is documented as a potent activator of both CD8^+^T cells and NK cells through the binding to the heterotrimeric receptor consisting of three subunits including α, β, and γ.^367,368^ In 1998, high-dose IL-2 was approved for melanoma treatment and obtained a considerable objective response in 15–20% patients with advanced melanoma.^369^ However, the increased risk of severe adverse effects including capillary, leak syndrome, gastrointestinal side effects, fever and chills limited the continuous usage of IL-2 to prolong the survival of patients. Other interleukins like IL-15 and IL-10 also have immune stimulatory function and antitumor capacity.^370–372^ Novel IL-15 super agonist complex and PEGylated formulation of recombinant IL-10 have been processed to clinical trials for treating melanoma, revealing promising therapeutic effect and satisfying tolerance.^373,374^

Inflammasomes are a class of cytosolic multiprotein complexes classically consisting of the NOD-like receptor (NLR) sensor protein, the adaptor protein ASC and the downstream effector caspase-1.^375^ Generally, inflammasomes act as a sensor and responder to pathogen-associated molecular patterns (PAMPs) via the maturation of pro-inflammatory IL-1β and IL-18 to activate immune cells, thus defending against various pathogens.^376^ For melanoma, Okamoto et al. firstly unveiled that NLR family pyrin domain-containing 3 (NALP3) inflammasome is constitutively assembled and activated, which is responsible for the spontaneous secretion of IL-1β from melanoma cells. The increased IL-1β could promote angiogenesis and modulate immune cells to promote melanoma progression.^377^ Bioinformatics analysis of pan-cancer data further demonstrates that NLRP3 inflammasome gene signature could be regarded as an independent prognostic factor of melanoma, with better predictive credibility than either tumor mutation burden (TMB) or tumorous glycolytic activity.^378^ Through a series of functional and mechanistic studies, Tengesdal et al. has proved that tumor-associated NLRP3/IL-1β signaling promotes the expansion of myeloid-derived suppressor cells (MDSCs), thereby resulting in ameliorated natural killer and CD8^+^ T cell activity and increased presence of Treg cells in tumor microenvironment. The combination of NLRP3 inhibition and anti-PD-1 antibody has obtained a synergized effect via the suppression of the function of MDSCs.^379^ In parallel with this, the activation of NLRP3 is also responsible for the role of tumorous PD-L1 in the resistance to anti-PD-1 immunotherapy through the regulation of MDSCs.^380^ In aggregate, inflammasomes play an oncogenic role in melanoma via the simultaneous effect on both tumor cell behavior and antitumor immunity. Targeting inflammasome is a promising strategy as a monotherapy or a combined option with immunotherapy for melanoma.

Angiogenesis is defined as the formation of new blood vessels derived from pre-existed neoplastic vasculature, which is mainly responsible for supplying sufficient nutrient and oxygen to ensure the rapid proliferation of tumor cells in cancer carcinogenesis.^381^ The first piece of evidence on angiogenesis in melanoma is the increased blood supply after the transplantation of melanoma cells into the cheek pouches of hamsters discovered by Warren et al.^382^. Then, the status of angiogenesis is found to be highly correlated with melanoma progression, especially the transition from the radial growth phase to the vertical growth phase.^383^ The development of a rich vascular architecture within the tumor microenvironment is orchestrated by angiogenic switch, which means the potentiation of pro-angiogenic factors and the suppression of anti-angiogenic factors.^384^ There are several main factors contributing to neovascularization in melanoma, including VEGF-A, basic fibroblast growth factor (bFGF), placental growth factor (PlGF), angiopoietin (Ang), IL-8, and PDGF. These factors are produced mainly by tumor cells, with alternative types of cells like endothelial cells and immune cells also participating in.^385^ To be specific, VEGF-A secreted from melanoma cells exerts its effect on angiogenesis through the binding to the receptor VEGFR on endothelial cells. Consequently, nitric oxide synthase (NOS) and PI3K/AKT signaling are activated to increase the permeability of vessel and promote endothelial cell proliferation and tube formation.^386,387^ In addition, the resultant activation of downstream focal adhesion kinase (FAK) contributes to melanoma cell extravasation across the vessel barrier.^388^ Similar to other cancers, the expression level of VEGF-A in melanoma is also under the transcriptional regulation of HIF1α, which is induced by local tissue hypoxia if the pace of angiogenesis does not meet the demand of nutrient and oxygen required for tumor cell proliferation.^389^ Apart from VEGF-A, bFGF is another principle pro-angiogenesis factor that is physiologically implicated in the modulation of wound healing via stimulating the proliferation of endothelial cells and the migration of macrophages and fibroblasts.^385^ Mechanistically, bFGF secreted from melanoma cells could not only interact with the receptor on the surface of endothelial cells to facilitate neovascularization, but also activate tumor cells in an autocrine way to promote their proliferation.^381^ The suppression of bFGF activity through the employment of targeted antibody or antisense oligodeoxynucleotides leads to prominent regression of angiogenesis and diminishment of tumor growth.^390,391^ Therefore, bFGF is a multi-effect therapeutic target for melanoma treatment. What’s more, PIGF, as a member of the VEGF family, has also been documented as a crucial promoter of angiogenesis in melanoma. Aside from the canonical receptor NRP-1 and NRP-2, PIGF can also bind to VEGFR via the formation of heterodimers with VEGF-A, so that the similar downstream pathways responsible for angiogenesis would be activated as that of VEGF-A alone.^392^ PlGF is also capable of directly interacting with VEGFR-1-positive hematopoietic precursors and pericytes and smooth muscle cells as well, so as to increase the recruitment and migration of hematopoietic precursors from bone marrow and promote the maturation of newly formed blood vessels as well.^392^ The above-mentioned mechanisms indeed mediate PIGF-driven melanoma growth and metastasis in a transgenic mice model in vivo, indicating that PIGF is of high potential as a therapeutic target in inhibiting melanoma progression via the obstruction of angiogenesis.^393^ Additionally, mounting evidence also reveals that other pro-angiogenic factors Ang, IL-8, and PDGF play a crucial role in facilitating the establishment of neovascularization and melanoma progression through multiple downstream mechanisms.^394–400^ In aggregate, the pro-angiogenic mechanism in melanoma is of rather complexity and highly interconnected due to the co-existence of multiple stimulatory ligands and receptors that exert their function through both paracrine and autocrine manners, which might be the reason for the limited therapeutic efficacy of treatment with a monoclonal antibody neutralizing VEGF-A in melanoma.^401^ Therefore, simultaneous suppression of multiple angiogenic pathways may be more effective.

## Current progresses in targeted therapy in melanoma

### MAPK inhibition-targeted therapy

The identification of *BRAF* mutation in melanoma in 2002 has opened a new era for understanding oncogenic events of melanomagenesis and provided the molecular basis for developing targeted therapy.^25^ Over 50% of cutaneous melanomas harbor *BRAF* mutation, which can induce a robust increase of its kinase activity and constitutive enhancement of downstream MEK-ERK signaling cascade.^402^ About ten years ago, vemurafenib and dabrafenib had been approved by FDA for the treatment of advanced melanoma harboring *BRAF* mutations. As a result, the two BRAF-targeted agents achieve a considerable objective response rate and some patients can even gain complete regression of tumor.^20,403^ Vemurafenib was the first-in-class agent which provided dramatic improvement of treatment outcome. Compared with dacarbazine chemotherapy in the phase III BRIM3 trial, vemurafenib single-agent treatment significantly increased the objective response rate (ORR) from 5 to 48%.^404^ In addition, the median progression-free survival (PFS) and overall survival (OS) were also substantially extended to 5.3 and 13.3 months respectively.^405^ The second BRAF-targeted agent dabrafenib was then developed which exhibited similar therapeutic effects as that of vemurafenib, with an objective response rate of 50% and progression-free survival being 5.1 months.^19^ It should be noted that the application of BRAF-targeted agent can induce the onset of keratoacanthoma and squamous-cell carcinoma, in around 15–20% of patients.^19,405^

Given the molecular rationale that *BRAF* mutation robustly triggers the hyper-activation of downstream MEK-ERK pathway, MEK-targeted agent trametinib has then been developed for melanoma targeted therapy. According to the result from the phase III METRIC trial, trametinib single-agent treatment obtained an ORR of 22% and a median PFS of 4.8 months,^406^ which was not as ideal as that of BRAF-targeted therapy in melanoma harboring *BRAF* mutation. The application of MEK-targeted agent has been then extended to melanomas lacking *BRAF* mutation, since that some other populations of melanomas are also dependent on MAPK signal pathway to survive and growth. To be specific, *NRAS*^Q61R^-mutant melanoma receiving trametinib single-agent treatment obtained an ORR of 20% and a median PFS of 4 months in a phase II study.^407^ Forwardly, MEK inhibitor monotherapy has been approved in non-*BRAF*-mutation melanoma settings based on the results of phase III study, whereas the effect was not that satisfactory. Therefore, the combinations with CDK4/6 inhibition, MDM2 inhibition, and PI3K/AKT-pathway inhibition based on some mechanistic insights has been evaluated in recent studies.^408–410^

For patients with *BRAF*-mutant melanoma receiving MAPK inhibition-targeted therapy, drug resistance would inevitably occur within 6–12 months, which significantly hinders the treatment efficacy and result in frequent recurrence.^406^ Based on the distinct mechanisms and characteristics, resistance to MAPK inhibition therapy is classified to three types including intrinsic resistance, adaptive resistance and acquired resistance. To be specific, intrinsic resistance refers to the innate capacity of tumor cells to resist the toxicity of targeted inhibitors, which is decisive for the innate response to treatment. However, in the early phase after the application of targeted therapy, especially the first 24–48 h, multiple protective signaling pathways would be rapidly activated to mitigate the pro-apoptotic effect of targeted agent, which is defined as adaptive resistance. Later on, long-term MAPK inhibition treatment can lead to intracellular mutational alterations and establishment of mutational clones, contributing to the acquired resistance that is irreversible.^78^

### Signal pathways of resistance to MAPK inhibition-targeted therapy

Approximately 20% of melanoma patients are intrinsically insensitive to targeted therapy even their tumors harbor *BRAF* mutation. Previous investigations have demonstrated multiple genomic and non-genomic alterations rendering intrinsic resistance, including loss of *PTEN*, loss of *NF1*, *CCND1* amplification, COT upregulation, *RAC1* mutation, eIF4F activation, low MITF expression, and high AXL expression.^411,412^ Mechanistically, deletions or mutations of *PTEN* can trigger the activation of AKT signaling and thereby the suppression of downstream pro-apoptotic signaling.^413^ Melanoma cell lines with loss of *PTEN* are more resistant to BRAF inhibitor.^413^ More importantly, patients with melanoma carrying wild-type *PTEN* are reported to have better survival after targeted therapy.^414^ Besides, loss of *NF1* gene that encodes neurofibromin can result in the activation of downstream RAS, PI3K-AKT-mTOR and MAPK pathways through multiple mechanisms, so as to defend against the inhibition of MAPK in response to targeted therapy.^415,416^ *RAC1* mutation is also documented responsible for downstream RAS activation that underlies the intrinsic resistance to BRAF inhibition.^417^ In a previous clinical study enrolling 45 patients receiving the treatment with BRAF inhibitors, three out of them who had *RAC1* mutation revealed no prominent response. The deficiency of RAC1 can amplify the inhibitory effect of BRAF-targeted agent on melanoma cell survival.^417^ In addition, low MITF/AXL ratio that indicates the phenotypic plasticity of melanoma cells is reported to determine the intrinsic response to targeted therapy. Drug cocktails containing AXL inhibitor can promote melanoma cell elimination by MAPK inhibition.^93^

During early phase after targeted therapy, intracellular protective signaling pathways are activated to enable tumor cell survival. The establishment of adaptive resistance not only impairs the therapeutic efficacy, but also offers sufficient time to develop acquired resistance.^78^ Therefore, it is necessary to restrain adaptive resistance so as to delay or block the occurrence of irreversible acquired resistance. Multiple mechanisms are responsible for this process, including resetting of ERK1/2 pathway activation, upregulation of RTKs, MITF upregulation, and metabolic rewiring. To be specific, BRAF-targeted inhibitors would downregulate the expressions of sprouty RTK signaling antagonist 2/4 (SPRY2/4) and dual-specificity phosphatases (DUSPs), which leads to the relief of the feedback suppression on Ras and reactivation of the ERK signaling, thus rendering the treatment resistance.^415,418^ Besides, adaptive upregulation of multiple RTKs including Erb-B2 receptor tyrosine kinase 3 (ERBB3), PDGFR, EGFR, and FGFR contributes to cell survival and protects tumor cells from apoptosis induced by BRAF inhibition.^419–421^ A recent study has revealed that it is the downregulation of SOX10 that is responsible for the upregulation of PDGFR and EGFR via the potentiation of TGFβ signal. Meanwhile, the upregulation of ERBB3 after BRAF inhibitor treatment is highly attributed to FOXD3-mediated transcriptional activation.^421^ What’s more, canonical melanocytic lineage-specific transcriptional factor MITF is also reported to be induced and to promote downstream PGC1α-dependent oxidative phosphorylation.^200,422^ Therefore, the suppression of MITF and mitochondrial function could be promising in overcoming adaptive resistance of melanoma cells to targeted therapy.

After long-term treatment with MAPK inhibition agents, intracellular mutational alterations would occur to promote the establishment of mutational clones that are irreversibly resistant to targeted therapy. There are a considerable number of alterations of signaling pathways enriched mainly in MAPK signaling, PI3K/AKT signaling and PDGF signaling that mediate this process.^423^ In particular, the occurrence of *RAS*, *MEK,* and *NF1* mutations, the amplification of BRAF, the upregulation of COT1 and the alternative splicing of *BRAF* mutation all contribute to hyper-activation of MAPK cascade,^420,424–428^ which can impede the suppression of MAPK pathway by BRAF inhibitor. Moreover, IGF-1R upregulation, *PTEN* loss, *PIK3CA* missense mutation, and *AKT* mutation all prominently promote the activation of pro-survival PI3K-AKT pathway, giving compensatory protective action upon the blockade of MAPK pathway.^413,428–430^ What’s more, the upregulation of EGFR and PDGFRβ has also been documented as a crucial mechanism that activated downstream protective factors to enable tumor cell survival.^75,420^ In particular, whole-exome sequencing (WES) of melanomas with acquired resistance to BRAF inhibitors has revealed that *BRAF* gene amplification is identified in around 20% of patients, leading to robust upregulation of BRAF protein expression and the reactivation of ERK in response to BRAF inhibition.^426^ Besides, p61BRAF^V600E^ splice variant is identified in a subgroup of patients with acquired resistance, resulting in the expression of truncated BRAF proteins that lack the N-terminal RAS-binding domain whereas keep the kinase domain. This alteration helps to form homodimers that is resistant to BRAF inhibitor treatment.^431^ These reports have pointed out the great implication of *BRAF* amplification and splicing in acquired resistance. Additionally, the activation of YAP/TAZ pathway also renders acquired resistance to targeted therapy via the transcriptional activation of cell-cycle facilitators.^432,433^ Downregulation of dual-specificity MAPK phosphatases and ring finger protein 125 (RNF125) can mediate acquired resistance to BRAF inhibitor via the activation of MER-ERK signal pathway and EGFR respectively.^434,435^ Of note, the signaling pathways contributing to acquired resistance are quite complex, indicating the intricate molecular network in mutational clones induced by prolonged treatment with MAPK inhibition agent.^423^ Therefore, to overcome the acquired resistance through the usage of single inhibitory agent targeting one specific pathway may be difficult to obtain an extensive benefit with broad coverage.

### Combinatorial MAPK inhibition-targeted therapy and alternative targeted agents

The inevitable occurrence of resistance to single-agent targeted therapy has prompted the development of combinatorial regimens. Since that a series of mechanisms facilitate MAPK pathway hyper-activation and make melanoma cells refractory to sole BRAF inhibitor, combined inhibition of MEK is subsequently evaluated, and encouraging outcomes have been obtained in two phase III clinical trials COMBI-v and COMBI-d.^436,437^ Compared to previous single BRAF-targeted therapy, combined inhibition of both BRAF and MEK lead to the upregulation of clinical response rate from 50% to 60–70%. In addition, the profile of adverse effects is also changed in these two different therapeutic paradigms. The incidence of keratoacanthoma and squamous-cell carcinoma is significantly reduced in combinatorial group compared with BRAF inhibitor single-agent group. After the treatment with vemurafenib or dabrafenib, approximately 15–30% of total patients would develop keratoacanthoma and squamous-cell carcinoma, which is due to the acquisition of *RAS* mutations and paradoxical activation of CRAF in keratinocytes.^438,439^ Therefore, based on improved treatment outcome and mitigated adverse effects, the combinatorial therapy dabrafenib plus trametinib has been approved by FDA for treating patients with unresectable or metastatic melanoma harboring *BRAF*^V600E^ or *BRAF*^V600K^ mutations.^437,440^ Later on, the other two BRAFi/MEKi combinations including vemurafenib plus cobimetinib and encorafenib plus binimetinib have been also approved by FDA. Results from clinical trials have revealed that the therapeutic efficacy of these approaches are comparable,^21,440–442^ and indirect side-by-side analysis also supports this conclusion.^443^ The development of these combinations provides more options for patients with advanced melanoma harboring *BRAF* mutation.

It has been reported that various alterations like *RAS* mutations, feedback reactivation of receptor tyrosine kinases and RAS, BRAF amplification and BRAF^V600E^ splice variants are responsible for acquired resistance to BRAF-targeted therapy by facilitating the dimerization of RAF proteins and subsequent activation of ERK signal,^420,426,430,444^ causing the so-called paradoxical effect. Therefore, next-generation RAF inhibitors proposed as pan-RAF inhibitors have been developed to obtain the equipotent suppression on both of RAF monomers and dimers,^445–448^ which is different from vemurafenib or dabrafenib that could only suppress one protomer within the RAF dimer. These pan-RAF inhibitors like TAK-632, LY3009120 and AZ-628 favor catalytic inhibition of both RAF protomers within the dimer via the stabilization of the αC-helix toward the active IN position.^447,449,450^ Ongoing clinical trials have revealed that these agents are well-tolerant, but can also disturb the aberration of essential MAPK signal in normal tissues and induce additional AEs.^451^ Of note, due to the lack of selectivity toward the suppression of mutant RAF dimers in cancer cells compared to the inhibition of wild-type RAF dimers in normal cells, LY3009120 showed limited efficacy at its maximum tolerated dose,^452^ which prompts the generation of selective RAF dimer inhibitors to suppress resistant RAF dimers in tumors more effectively.

Moreover, paradox breakers that are a class of BRAF inhibitors have been recently developed by Plexxikon, aiming to obtain the inhibition of ERK1/2 in BRAF^V600E^ cells without driving paradoxical activation of ERK1/2 in *RAS*-mutant cells.^453^ After the investigation of hundreds of vemurafenib derivatives for biochemical activity and cell activity, two chemicals named PLX7904 and its further optimized analog PLX8394 are found to be capable of evading the paradoxical MAPK pathway activation. To be specific, these two drugs can directly suppress the formation of BRAF:CRAF heterodimers normally observed in *RAS*-mutated cells receiving the treatment of RAF inhibitors, meanwhile maintaining the binding affinity for the dimer partner. Not surprisingly, PLX8394 now has entered the clinical trial stage (ClinicalTrials.gov identifiers: NCT02012231 and NCT02428712).^453^

Aside from BRAF and MEK inhibitors, alternative molecular targets and related agents have also been investigated in preclinical studies and clinical trials, especially ERK and AKT. In 2013, Morris et al. has identified a selective inhibitor of ERK1/2 called SCH772984 that can eradicate tumor cells harboring *BRAF*, *NRAS,* or *KRAS* mutation within nanomolar concentration. The treatment effect of SCH772984 on melanoma has been well confirmed in preclinical xenograft tumor model.^454^ Besides, the combination of vemurafenib and SCH772984 has been proved to have synergistic effect on suppressing *BRAF*-mutant melanoma progression, which can help to delay the onset of the resistance to targeted therapy.^455^ In this case, SCH772984 could be broadly used in a wide variety of melanomas of distinct genetic backgrounds, only if they manifested hyper-activation of ERK signaling. What’s more, the frequent occurrence of AKT hyper-activation and its crucial tumorigenic role in melanoma have encouraged the investigations of targeting AKT in treating melanoma. GSK2141795 has been discovered as an orally bioavailable and highly potent AKT-specific inhibitor, which exhibits prominent antitumor effects in combination with the MEK inhibitor.^456^ Of note, the initial combination of AKT inhibitor GSK2141795 and MAPK inhibitors revealed superior growth inhibitory effects compared with the later addition of AKT inhibitors to tumors with acquired resistance to MAPK inhibitors.^457^ However, dual MEK/AKT inhibition with Trametinib and GSK2141795 did not yield clinical benefits in metastatic *NRAS*-mutant and wild-type melanoma,^458^ indicating the limited spectrum of application of AKTi in melanoma with rather high heterogeneity. What should also be noticed is belvarafenib, a potent and selective RAF dimer (type II) inhibitor, exhibits clinical efficacy in patients with BRAF^V600E^ and *NRAS*-mutant melanomas. The first-in-human phase I study of belvarafenib for melanoma treatment has been conducted (NCT02405065, NCT03118817). In addition, by generating belvarafenib-resistant *NRAS*-mutant melanoma cells and analyzing circulating tumor DNA from patients treated with belvarafenib, new recurrent mutations in *ARAF* have been identified to confer the treatment resistance. The combination of RAF plus MEK inhibition may be used to delay ARAF-driven resistance.^459^

## Present advances in therapies targeting tumor immunology and ongoing clinical trials

### Immune escape and related signal pathways

Apart from directly targeting driver genes that contribute to tumor intrinsic malignancy, therapies targeting the immune system, which is generally thought to eliminate cancer cells, have shown promising efficacy in melanoma treatment. The importance of the host immune system in eradicating cancer cells has long been appreciated, with instances of spontaneous melanoma regression.^460^ Thomas and Burnet initially introduced the notion of immunosurveillance, demonstrating that the immune system is responsible for eliminating malignant cells via recognition of tumor-associated antigens,^461^ and it was further experimentally confirmed by the observation of increased incidence of melanoma in immune-deficient patients.^462^ However, immunosurveillance is only one part of the complicated interplay between cancer and host immune system. The relationship between cancer and immune cells is composed of three phases: elimination, equilibrium, and escape.^462^ Elimination indicates a classical view of immunosurveillance. During the early stage of tumorigenesis, the innate immune cells present tumor antigens through binding to MHC and activate adaptive immune cells via co-stimulatory signal, as well as releasing cytokines to eradicate cancer cells. However, the plasticity of melanoma enables transformed tumor cells to evade immunosurveillance,^463^ indicating the relationship between the two moving to the next phase, equilibrium. In this stage, the host immune system could control the outgrowth of tumor through eliminating immune-sensitive tumors. The last phase, escape, refers to the outgrowth of tumors that have got rid of the restrain of immune system.

Although melanoma represents one of the most immunogenic tumors, which could have elicited adaptive antitumor immune response due to its high mutational burden,^464^ yet plasticity of melanoma allows it to evade the immunosurveillance of the immune system.^465,466^ Mechanisms involved in tumor immune escape mainly include: developing lesions in antigen processing, increased resistance to cytotoxicity induced by immune cells and development of immunosuppressive tumor environment.^467–469^ Tumor cells escape T cell recognition through downregulation of tumor antigens and MHC, as well as impaired antigen processing. On the other hand, immunosuppressive cytokines, such as TGF-β and granulocyte-macrophage colony-stimulating factor (GM-CSF), were released to facilitate the recruitment of MDSCs or the suppression of antigen-presenting cells (APCs). Moreover, melanoma cells recruit immunosuppressive regulatory Treg cells through secreting relevant chemokines, and inhibit the natural cytotoxicity of NK cells against tumor cells through metabolic reprogramming.^470,471^ Last but not the least, melanoma cells directly induced the exhaustion of cytotoxic T cells by expressing co-inhibitory molecules PD-L1, which binds to the co-inhibitory receptor PD-1 located on the surface of T cells.^472–474^ Recently, by linking the antigenic specificity of T cell receptors (TCRs) and the cellular phenotype of melanoma-infiltrating lymphocytes at single-cell resolution, the interplay between tumor cell phenotypic characteristics and TCR properties is revealed. Melanoma-reactive lymphocytes predominantly displayed an exhausted state that encompassed diverse levels of differentiation but rarely acquired memory properties, suggesting that tumor specificity shapes the expression state of intra-tumoral CD8^+^T cells.^475^ The targets of immunotherapies in the treatment of melanoma mainly focus on the mechanisms associated with the formation of an immunosuppressive environment.

### The era of therapies targeting tumor immunology

#### Cytokines

Since the 1950s, immunotherapy has been an appealing area of cancer treatment research, and attempts to reactivate immune response against tumor cells have been made. However, “Coley’s toxin”^476,477^ and immune stimulants, such as Bacillus Calmette Guerin,^1^ which meant to augment non-specific immune responses, have all failed to show significant response in melanoma.^423^

IFNα-2b, a member of the type I interferon family, demonstrates multiple antitumor activities including the suppression of proliferation and angiogenesis, and the enhancement of antitumor immune response through increasing the cytotoxicity of NK cells and tumor antigen processing.^478^ In 1995, high-dose IFNα-2b became the first exogenous cytokine approved by FDA for the adjuvant treatment of advanced melanoma.^479^ Over a decade later, Pegylated IFNα-2b, polyethylene glycol-modified IFNα-2b with a longer half-life also obtained FDA approval, although still with low efficacy and sever toxicity.^423,480^ Gao et al. found that another type I interferon member, IFNα-1b, which was approved by Chinese Food and Drug Administration for cancer treatment, possessed improved safety and efficacy especially in patients with unresectable metastatic melanoma.^365,423^

IL-2 is the second cytokine approved by FDA in 1998 for the use of patients with stage IV melanoma.^481^ As a cytokine, IL-2 could activate antitumor response of cytotoxic T cells and NK cells. Although high-dose IL-2 shows promising results in melanoma with an 18% objective response rate, the severe side effects and low response rates of the regimen restrict the use of it in larger population.^481–483^ Attempts to improve response rate of high-dose IL-2 have been made by evaluating the efficacy of high-dose IL-2 combined with IFNα, which, however, showed a minimally improved ORR of 25%.^484^ Since the severe toxicity of high-dose IL-2 and IFN-γ leads to the intolerance of patients to treatment and restrains the therapeutic efficacy in a large population, the combination of chemotherapies, targeted therapies or other immunotherapies with low-dose IL-2 or IFN-γ has been employed in some investigations to avoid the side effects brought by single high-dose cytokines therapy, which can meanwhile achieve better treatment response.^481,485–488^ The reasons for relatively low response rate of IL-2 probably attributed to the activation of immunosuppressive Tregs.^489^ Based on this, many modified forms of IL-2, including PEGylation form, antibody-cytokine conjugates, and fusion proteins began to emerge, aiming to extend the half-life of IL-2 and elevate the ORR of patients through inhibiting the binding of IL-2 to CD25 (receptor of IL-2) that lead to the activation of Tregs.^490^ A preclinical study has revealed that engineered IL-2, a CD25-mimobody, shows lower toxicity and increased potency to IL-2, and it still needs further assessment as a therapeutic agent.^491^

#### Oncolytic virus

Oncolytic virus therapy, a new class of antitumor immunotherapy, can lead to tumor regression through selective kill of tumor cells, induction of immunogenic cell death, and stimulation of systemic antitumor immune response.^492^ Until recent years, clinical benefits have been observed with intra-tumoral administration of oncolytic virus. In 2015, FDA approved the first oncolytic virus therapy, T-VEC for the local treatment of patients with recurrent melanoma that cannot be surgically removed, based on results from a phase III study of patients with metastatic melanoma lesions in skin and nodes. T-VEC is a genetically modified herpes simplex virus type 1 (HSV-1) with reduced virulence and selective proliferation in tumors, encoding GM-CSF, which promotes the priming of T cell responses.^493^ A randomized open-label phase III clinical trial evaluating the efficacy and safety of T-VEC compared with GM-CSF revealed promising durable clinical benefits with a higher ORR (31.5% vs 6.4%), higher durable response rates (19.0% vs 1.4%), and longer median OS (23.3 months vs 18.9 months) versus GM-CSF alone, as well as favorable safety profile, especially in IIIB-IVM1a melanoma patients.^494,495^ Subsequent multi-center studies also demonstrated high rates of complete and durable response in advanced melanoma patients administrated with T-VEC,^496,497^ even for the local lesions of patients who developed acquired resistance to immune checkpoint blockade.^498^

#### Therapies targeting immune checkpoints

In the last few decades, immune checkpoint blockades (ICBs) therapy has led to important clinical advances, which holds great promise in cancer treatment. Normally, T cell activation requires two signals upon recognition of tumor antigen presented on the surface of APCs. TCR specifically binds to an antigen in the context of MHC and a co-stimulatory signal transduced by CD28 on T cell surface which could be stimulated by B7 molecules (CD80 and CD86) on the APCs.^499,500^ The co-receptors on the T cells engaged in secondary signal could be stimulatory or inhibitory. Co-stimulatory molecules such as CD28 and B7 mediate T cell activation, while co-inhibitory molecules including PD-1, CTLA-4, PD-L1, and PD-L2, which are known as “immune checkpoints”, function as T cell brakes.^501,502^ To date, the most well-studied immune checkpoints are CTLA-4, PD-1, and PD-L1, the blockade of which has shown promising effects against various cancers through reinvigorating antitumor immunity.^503–508^

Upon T cell activation, CTLA-4 expression is initiated which could bind to B7 molecules with much higher affinity than CD28, resulting in an inhibited immune response.^509–511^ Preclinical studies have demonstrated that CTLA-4 blockade with an antagonistic antibody leads to improved T cell function and regression of tumor cells in mouse models.^512–514^ Ipilimumab, a fully human monoclonal antibody against CTLA-4, is the first approved immune checkpoint by the FDA for the use in patients with advanced melanoma in 2011.^515,516^ Although only a small part of melanoma patients benefits from ipilimumab treatment and noticeable side effects associated with immune-related adverse events (irAE) could occur, patients with unresectable advanced melanoma treated with ipilimumab have a long-term survival effect.^517–519^

PD-1 is another co-inhibitory receptor expressed on the surface of T cells upon T cell activation, which could bind to its receptor including PD-L1 and PD-L2, on the surface of tumor cells or other immune cells within the tumor environment to control the cytolytic function of effector T cells through activation of the tyrosine phosphatase SHP1/2 signaling.^24,520^ As such, antibodies against PD-1/PD-L1 are supposed to be effective for the treatment of cancers via the invigoration of infiltrating CD8^+^T cells.^521^ Pembrolizumab and nivolumab are the two PD-1 inhibitors approved by FDA in 2014. Both nivolumab and pembrolizumab demonstrated improved survival benefits versus ipilimumab^522–526^ and chemotherapies, such as dacarbazine,^527–529^ with a prolonged progression-free survival and overall survival and elevated response rates. Clinical trials have revealed the response rate for ipilimumab-refractory patients treated with nivolumab or pembrolizumab was 30%, and 1-and 2-year survival rates were 68.4% and 31.2%.^530,531^ Due to the improved efficacy and much more tolerable toxicity of nivolumab and pembrolizumab than that of ipilimumab, PD-1 inhibitors have become the first-line treatment of patients with BRAF-wide-type metastatic melanoma.^532^ Nivolumab and pembrolizumab have also shown clinical benefits in patients with other types of melanoma including untreated melanoma brain metastases,^533–535^ uveal melanoma,^536^ acral lentiginous melanoma and mucosal melanoma,^537^ resulting in 46%, 19%, 18.8%, and 20.8% ORR, respectively. In addition, pembrolizumab has shown antitumor activity in aged melanoma patients,^538^ but not in pediatric melanomas.^539^ Toripalimab, a selective recombinant human PD-1 monoclonal antibody, has been approved by China FDA in 2018 for the treatment of a variety of cancers, including melanoma.^540^ Treatment with toripalimab resulted in an ORR of 20.7% in patients with advanced melanoma, most of which are acral and mucosal melanoma.^540–542^ Base on the promising clinical results seen with toripalimab-treated mucosal melanoma, FDA has granted toripalimab a fast track designation for use in the frontline treatment of patients with mucosal melanoma. HX008, another humanized IgG4 monoclonal antibody against PD-1 showed favorable clinical benefits for Asian melanoma patients, who had been treated with chemotherapy, targeted therapy, or immunotherapy, with an ORR of 20.2% according to the results from a phase II study.^543^

Given that PD-1 and CTLA-4 participate in different processes of T cell recognition and cytotoxic T cell reinvigoration, combinations of antibodies against PD-1 and CTLA-4 are of great potential to induce tumor regression synergistically. Clinical trials of combined nivolumab and ipilimumab versus nivolumab or ipilimumab monotherapy in previously untreated melanoma have revealed that patients treated with nivolumab plus ipilimumab obtained durable and sustained clinical benefits.^544–547^ The 3-, 4- and 5-years overall survival rates were 58%, 53%, and 52% for the patients treated with nivolumab and ipilimumab, 52%, 46%, 44% for the nivolumab group, as compared with 34%, 30%, 26% for the ipilimumab group, respectively.^525,548,549^ Based on the results of the trials, FDA-approved combined nivolumab and ipilimumab for the frontline use of patients with advanced melanoma in 2015. However, the combination of nivolumab and ipilimumab also leads to increased treatment-related adverse events compared with nivolumab and ipilimumab (59% vs 23% and 28%).^548^ A subsequent clinical trial was conducted in melanoma patients with sequential administration of nivolumab and ipilimumab as well as the reverse sequence, and demonstrated that nivolumab followed by ipilimumab have a lower toxicity and similar clinical benefits with co-administration of nivolumab and ipilimumab.^550^ Studies of modulated dosing regimen for nivolumab plus ipilimumab trying to decrease the toxicity display a lower incidence of treatment-related adverse events without weakening the antitumor activity for the treatment of higher/standard-dose nivolumab and a lower-dose ipilimumab.^551^ Of note, improved antitumor response and tolerable safety are seen in anti-PD-1/PD-L1-refractory melanoma patients with pembrolizumab plus low-dose ipilimumab.^552^ The treatment of other types of melanoma excluding cutaneous melanoma is of great challenge for their relatively insensitive response to existing therapies. The combination of nivolumab and ipilimumab has been subsequently evaluated in non-cutaneous melanoma including acral, mucosal, and uveal melanoma, and the combination regimen shows sustained and improved response compared with either single agent, although with elevated toxicity but manageable safety profile.^553–555^ Clinical trials have also been conducted in another population of patients with brain metastasis of melanoma, which were generally excluded from clinical trials for their poor prognosis. In two phase II studies, combination of nivolumab and ipilimumab displays similar intracranial antitumor effects in a relatively small population.^534,556^ Another clinical trial of 380 patients with asymptomatic and symptomatic melanoma brain metastasis also shows that the 2- and 3-years overall survival rates are 41% and 30% with the combination regimen, respectively.^557^ The combination of another standard-dose anti-PD-1, pembrolizumab, and lower-dose ipilimumab provides robust clinical benefits with a 3-year ORR of 62.1%,^558,559^ while studies of standard-dose pembrolizumab plus alternate-dose ipilimumab shows that pembrolizumab 200 mg plus ipilimumab 50 mg could prominently reduce the toxicity of the combination therapy.^560^

PD-L1 and PD-L2 that are PD-1 ligands expressed on tumors cells contribute to cancer cell evasion. Most melanomas were reported to highly express PD-L1.^561^ Actually, PD-L1 is expressed in tumor cells and myeloid cells in the tumor environment mainly mediated by constitutive activation of oncogenic signal pathways in tumor cells and IFNγ signaling.^499,562^ The expression of PD-L2 is highly upregulated in certain B cell lymphoma.^501^ Given that PD-1 binds to both PD-L1 and PD-L2, and PD-1 interacts with either CD80 or PD-L1, the antibodies targeting PD-1 or PD-L1 may lead to different antitumor effects and toxicities,^563^ which has been supported by relevant clinical trials of anti-PD-L1. Although single use of antibodies against PD-L1 such as durvalumab, avelumab, and atezolizumab has been approved for the use in patients other than melanoma, yet investigations of PD-L1 antibodies in combination with targeted therapy or/and chemotherapy are still underway in the treatment of melanoma. Notably, FDA has approved atezolizumab for unresectable or metastatic melanoma harboring *BRAF* mutation in combinatorial regimens with targeted therapies.

#### Adjuvant and neoadjuvant therapy

For advanced melanoma patients with high risk of recurrence, adjuvant therapy is offered to lower the risk of recurrence after the surgery. High-dose IFNα-2b of pegylated IFN had been the sole approved adjuvant therapy in the treatment of melanoma for a long time. However, adjuvant IFNα-2b showed marginally significant and slightly diminished positive effects on the recurrence-free survival (RFS) and OS of resected stage III melanoma patients, concomitant with intolerable toxicity.^564,565^ In 2005, adjuvant ipilimumab at a dose of 10 mg/kg resulted in significantly higher rates of RFS and OS, although with higher rates of immune-related adverse events than with placebo, which led to the approval of adjuvant treatment of ipilimumab for stage III melanoma patients.^566^ A phase III clinical trial demonstrated that adjuvant ipilimumab (10 mg/kg) was not superior in efficacy to IFNα-2b, but ipilimumab (3 mg/kg) significantly improved the OS with lower toxicity compared with high-dose IFNα-2b.^567^ In 2017, adjuvant anti-PD-1, nivolumab, showed longer RFS and lower rates of high-grade adverse events than adjuvant with ipilimumab among patients with resected stage III or IV melanoma in a phase III trial, Checkmate 238.^568^ Comparisons of another anti-PD-1, pembrolizumab with placebo also showed improved efficacy and favorable safety profile.^569–571^ Based on those trials, nivolumab and pembrolizumab had been approved for adjuvant treatment of patients with unresected stage III melanoma. Clinical trials of combining nivolumab and ipilimumab or nivolumab monotherapy in patients with stage IV melanoma are still ongoing.^572^ Besides, combined targeted therapies like BRAF inhibitor dabrafenib plus MEK inhibitor trametinib have demonstrated improved RFS and tolerable toxicity in patients with stage III *BRAF*^V600R/K^-mutant melanoma compared with the adjuvant use of placebo.^573^ Both anti-PD-1 treatment and BRAFi plus MEKi therapy are the frontline options for adjuvant therapy. However, how to choose between the two still needs further investigations.

Although no neoadjuvant treatment has been approved by the FDA, neoadjuvant therapy appears promising in the treatment of patients with high-risk melanoma. Ongoing clinical trials of neoadjuvant ipilimumab plus nivolumab have showed high pathological response rates in patients with macroscopic stage III melanoma, and it needs further investigation to preserve efficacy and reduce toxicity.^574^

### Signal pathways of resistance to ICBs

Although therapies targeting immune checkpoints have achieved better outcomes in patients of a variety of cancer types, only a minority of patients obtains a durable benefit from the treatment of ICBs, and some initial responders even have their tumors progressing on after a period of response. Unveiling the mechanism underlying the patients who do not respond or sustainably respond to ICBs is appealing for scientists. Resistance to ICBs could be classified into two categories: primary resistance and acquired resistance. Primary resistance refers to patients who do not respond to the initial ICB therapy. Acquired resistance means the cases in which patients have response to ICB therapy initially, but have tumor progression after a duration of therapy.^575^ Investigations to find out possible predictors of response to immunotherapy blockade have revealed that PD-L1 expression, tumor mutational burden,^576,577^ tumor intrinsic oncogenes, such as IFN-γ, p53, and Wnt signaling,^578–581^ signatures of T cell dysfunction and antigen presentation expression,^582–586^ gut microbiota and its derived metabolites^587–589^ are all significantly associated with clinical benefit. PD-L1 expression has been shown to identify melanoma patients who are more likely to respond to PD-1 inhibitors.^590^ However, it is not recommended to take PD-L1 expression into account for treatment decisions because of the imperfect correlation between PD-L1 expression and clinical benefits from PD-1 inhibitors.^525,591^ Higher tumor mutational burden is associated with better response to ICBs, which is thought to enhance antitumor immune response through augmenting neoantigen formation. IFN-γ signaling plays a pivotal role in stimulating antitumor response mainly through activating cytolytic T cells and promoting tumor antigen presentation,^592^ and IFN-γ signaling profile is related to response to ICBs.^353^ Activation of Wnt or Braf signaling as well as loss function of PTEN partially mediates ICBs resistance. The relationship of host gut microbiome and resistance to ICBs is complex and not elucidated. High level of microbiome-derived metabolites, especially short-chain fatty acids, is reported to mediate the resistance to CTLA-4 antibody via restraining the function of DCs and T cells.^589^ However, mechanisms of acquired resistance are far from fully understood. Similar to primary resistance, defects in antigen presentation and IFN-γ signaling, neoantigen depletion, and anergic T cells in tumor all contribute to acquired resistance to ICBs therapies.^593^ To overcome the resistance to ICBs, substantial efforts have been made on combinatorial approaches to broaden the responders and lower the toxicities.

### Combinatorial therapies targeting tumor immunology and mutated driver genes

With the disclosure of mechanisms underlying cancer growth and interactions between cancer cells and tumor environment, therapies targeting different intra-and extra-tumor processes undoubtedly increased. The pluralistic targets in cancer treatment provide a great potential for combinatorial regimens. While monotherapy may not display an optimistic clinical benefit or safety profile, harnessing combination approaches to maximize the antitumor effects while minimizing toxicities seems to be a promising strategy for cancer treatment. There are many clinical trials of combinatorial regimens under development, and most of them are combining ICBs, anti-PD-1/PD-L1/CTLA-4, along with targeted therapy or other immunomodulatory approaches.

Recently, engineered forms of cytokines with lower adverse effects and enhanced efficacy in combination with ICBs have also raised great interest. NKTR-214 (Bempegaldesleukin), a pegylated IL-2, preferentially binds to CD122 other than CD25, which leads to enhanced activation of T cells and NK cells and reduced toxicity resulting from Tregs activation.^594^ NKRT-214 plus nivolumab is well tolerated and has promising clinical efficacy with an overall ORR of 59.5%.^595^ Combination of pembrolizumab and pegylated IFNa-2b shows improved response rates in advanced melanoma.^596,597^ The combination of ICBs and T-VEC is appealing for the reason that T-VEC could activate antitumor immune response through promoting the expression of IFN-γ and immune checkpoints such as PD-L1, as well as stimulating tumor antigen presentation.^598^ Patients with advanced melanoma treated with combination of T-VEC and pembrolizumab have an elevated ORR of 62%.^598^ Consistently, combination of T-VEC and ipilimumab shows greater antitumor activity in the treatment of advanced unresectable melanoma without additional safety concerns versus ipilimumab.^599,600^ Adoptive cell therapy (ACT) is also a promising therapy for the patients who are refractory or non-tolerant to current first-line therapies. With the development of cellular therapy, ACT could be divided into three types according to a different mechanism of action: isolated tumor-infiltrating T cells from resected tumor, T cells with engineered chimeric antigen receptors (CAR-Ts) and T cells with engineered T cell receptor (TCR-Ts).^601^ The initial study of ACT using high-dose IL-2 combined with autologous TILs expanded in vitro after the treatment of chemotherapy achieved 60% objective regression of melanomas.^602^ Systematic analysis of the efficacy of ACT with TIL plus high-dose IL-2 showed durable clinical benefits in the treatment of advanced melanoma.^487^ Notably, adoptive transfer of TILs has achieved an ORR of 24% in patients who are refractory to PD-1 antibody^603^ and showed antitumor effects in acral and mucosal melanoma patients.^604^ Recently, long-term follow-up of lifileucel (LN-144) cryopreserved autologous TIL therapy reveals a promising effectiveness, with an ORR of 36.4% in pretreated melanoma patients who failed on first-class targeted therapy or ICBs.^605^ A trial of adoptive transfer of TIL engineered with IL-12 has demonstrated antitumor activity but with high toxicities.^606^ Combining ACT with TIL and IFN-α provides better median OS and disease-free survival in Chinese resected stage III melanoma patients.^607^ Although MART1-specific TCR-Ts shows clinical potency for melanoma patients, the specific loss of MART1 of tumor cells renders low response rates of the therapy.^601^ CAR-Ts have been approved for the treatment of hematologic tumor, but it did not show the same antitumor effects, not to mention severer toxicities, in melanoma treatment.^608^

The improvement of targeted therapies and immune checkpoint inhibitors has shed a light on the treatment of melanoma patients. BRAF and MEK inhibitors displayed high ORR, yet only lower than half of patients with BRAF^V600^-mutated melanoma could obtain long-term benefits form BRAF inhibitors.^609^ The ORR of ICBs is relatively lower than targeted therapies, although ICBs provide durable responses.^610,611^ Given that complementary clinical profiles led by targeted therapies and ICBs, the proposal that combinatorial regimen of these two therapies might provide durable response and elevated ORR, as well as lower toxicity are of great interest. Ample evidence has proved that BRAF and MEK inhibitors could promote the priming and function of tumor infiltration T cells, facilitate antigen presentation and modulate the tumor environment to be harmful for tumor cells in mouse model and in vitro.^612–617^ However, the combination of CTLA-4 inhibitor, ipilimumab, with BRAF inhibitor, vemurafenib, or dabrafenib and trametinib fails for severe liver or gastric toxicity.^618,619^ Subsequent studies focused on the combination of BRAF/MEK inhibitors and PD-1/PD-L1 blockades, which have better tolerable safety profiles than CTLA-4 blockades. Clinical trials of the triple combination of PD-L1 antibody, atezolizumab, BRAF inhibitor, vemurafenib and MEK inhibitor, cobimetinib result in significantly prolonged progression-free survival compared with the combination of vemurafenib and cobimetinib (15.1 months vs 10.6 months), and manageable toxicity.^620,621^ Based on the favorable results of the triplet combination, the FDA-approved atezolizumab in combination with cobimetinib and vemurafenib for patients with BRAF^V600^ mutated unresectable or metastatic melanoma in 2020. A phase II study of pembrolizumab, dabrafenib, and trametinib in BRAF-mutant melanoma has revealed improved PFS and OS. A nearly 3 years follow-up suggested a 16.9-months median PFS of the treatment of triplet combination and 10.7-months of the treatment with dabrafenib and trametinib.^622^ Combination of an investigational PD-1 antibody, spartalizumab, dabrafenib and trametinib leads to an ORR of 78% in advanced BRAF-mutant melanoma, including 44% complete response, which suggests the triplet combinatorial regimen is promising, although in a relatively small population.^623^ With the successful trials of combined targeted therapies and ICBs, numerous studies of combinatorial therapies begin to emerge. Clinical trials of combining PD-1/PD-L1/CTLA-4 blockades with MAPK inhibitors, EGFR inhibitors or BRAF and MEK inhibitor are underway in BRAF-mutant and wide-type melanoma.^624–628^ Recent studies of combined VEGF inhibitor, apatinib, and an investigational humanized IgG4 monoclonal antibody against PD-1, camrelizumab demonstrates an ORR of 22.2% in advanced untreated acral melanoma patients,^629^ and longer follow-up time is needed to confirm the efficacy of the combinatorial regimen. A retrospective study evaluating the efficacy and safety of VEGF inhibitor, axitinib plus anti-PD-1 provides improved clinical benefits, with an ORR of 24.5%.^630^ The latest results of trials evaluating atezolizumab in combination with VEGF inhibitor, bevacizumab, for the use in patients with advanced mucosal melanoma shows promising benefit with an unconfirmed ORR of 42.9% in a relatively small population, and phase III trials with large population was needed to confirm the benefits.^631^

### Novel targeted therapies for re-activating antitumor immunity and ongoing clinical trials

As we learn more about the mechanisms underlying cancer evasion, other new inhibitors and stimulatory immune checkpoints regulating function of T cells have sprung out in recent years. Blocking antibodies specific for those inhibitory receptors are under investigations, although most of them are far from clinical application. The majority of ongoing investigations about newly-discovered immune checkpoints are combination therapies with PD-1 or PD-1 plus CTLA-4 antibodies to broaden the responders or reduce toxicities (Table 3).

**Table 3:** Ongoing mono- or combination clinical trials for melanoma treatment

Target	Agents	Combinations	Trial identifier	Phase	Type of tumor	Status and results
CTLA-4	Ipilimumab	Plus TLR9 agonist IMO-2125	NCT03445533	III	Anti-PD-1 refractory melanoma	Active, not recruiting
		Plus VEGFR antagonist bevacizumab	NCT00790010	I	Unresectable stage III/IV melanoma	Active, not recruiting
		Plus VEGFR inhibitor axitinib	NCT04996823	II	Advanced melanoma	Recruiting
		Plus multi-kinase inhibitor cabozantinib	NCT04091750	II	Advanced melanoma	Recruiting
		Plus CCR4 antagonist FLX475	NCT04894994	II	Advanced melanoma refractory to anti-PD-1/PD-L1 antibody	Recruiting
PD-1	Nivolumab	Plus multi-kinase inhibitor cabozantinib	NCT04091750	II	Unresectable advanced melanoma	Recruiting
		Plus HDAC inhibitor tinostamustine	NCT03903458	I	Refractory, locally advanced, or metastatic melanoma	Recruiting
		Plus encorafenib and binimetinib	NCT04511013	II	BRAF V600 mutant melanoma with brain metastasis	Recruiting
	Pembrolizumab	Plus dabrafenib and trametinib	NCT02130466	I/II	Advanced melanoma	Completed
		Plus encorafenib and binimetinib	NCT02902042	I/II	Unresectable/metastatic BRAF V600 mutant melanoma	Completed
			NCT04657991	III	Unresectable/metastatic locally advanced BRAF V600 mutant melanoma	Recruiting
		Plus HDAC inhibitor 4SC-202	NCT03278665	I/II	Anti-PD-1 therapy refractory/non-responding melanoma	Recruiting
	HX008	Monotherapy	NCT04749485	II	Locally advanced or metastatic melanoma refractory to the standard treatments	Active, not recruiting
		Plus anti-PD-L1 antibody LP002	NCT04756934	I	Advanced or metastatic melanoma who have failed previous anti-PD-1/PD-L1	Recruiting
		Plus oncolytic virus HG52	NCT04616443	Ib/II	Melanoma	Recruiting
	Camrelizumab/SHR-1210	Plus VEGF inhibitor apatinib	NCT03955354	II	Advanced acral melanoma	Recruiting
		Plus VEGFR antagonist bevacizumab	NCT04091217	II	Unresectable locally advanced or metastatic mucosal melanoma	Recruiting
	Spartalizumab	Plus dabrafenib and trametinib	NCT02967692	III	Previously untreated unresectable/metastatic BRAF V600 mutant melanoma	Active, not recruiting
PD-1 and CTLA-4	Nivolumab plus ipilimumab	Plus BRAF/MEK inhibitor, vemurafenib, and cobimetinib	NCT02968303	II	Unresectable/metastatic melanoma	Recruiting
		Plus dabrafenib and trametinib	NCT01940809	I	Unresectable/metastatic BRAF V600 mutant melanoma	Active, not recruiting
		Plus dabrafenib and trametinib	NCT02224781	III	Unresectable/metastatic BRAF V600 mutant melanoma	Recruiting
PD-L1	Atezolizumab	Plus cobimetinib	NCT03273153	III	Previously untreated advanced BRAF V600 wild-type melanoma	Completed
			NCT01988896	I	Locally advanced/metastatic solid tumors	Completed
	Avelumab	Plus other cancer immunotherapies	NCT02554812	II	Advanced tumors	Active, not recruiting
LAG-3	Relatlimab/BMS-986016	Plus nivolumab	NCT03470922	III	Advanced melanoma	Active, not recruiting
		Plus nivolumab	NCT04552223	II	Uveal melanoma	Recruiting
	IMP-321/Eftilagimod alfa	Plus pembrolizumab	NCT02676869	I	Unresectable/metastatic melanoma	Completed
TIM-3	TSR-022	Plus PD-1 inhibitor dostarlimab (TSR-042)	NCT04139902	II	Resectable regionally advanced or oligometastatic melanoma	Recruiting
	INCAGN02390	Plus anti-LAG-3, INCAGN02385 and anti-PD-1 INCMGA00012	NCT04370704	I/II	Advanced tumors	Recruiting
	LY3321367	Plus anti-PD-L1 antibody LY3300054	NCT02791334	I	Advanced refractory solid tumors	Active, not recruiting
TIGIT	Vibostolimab	Plus pembrolizumab	NCT04303169	I/II	Neoadjuvant therapy to advanced melanoma	Recruiting
		Plus pembrolizumab	NCT04305054	I/II	Advanced melanoma	Recruiting
		Plus pembrolizumab and anti-CTLA-4, quavonlimab	NCT04305041	I/II	PD-1 refractory melanoma	Recruiting
4-1BB	PF-05082566	Plus OX40 agonist PF-04518600	NCT02315066	I	Locally advanced/metastatic melanoma	Completed
		Plus CD20 antibody rituximab	NCT01307267	I	Advanced tumors	Completed
OX40	MEDI0562	Plus tremelimumab or durvalumab	NCT02705482	I	Advanced solid tumors	Completed
	INCAGN01949	Plus nivolumab or/and ipilimumab	NCT03241173	I/II	Advanced/metastatic tumors	Completed
	INBRX-106	Plus pembrolizumab	NCT04198766	I	Locally advanced/metastatic solid tumors	Recruiting
	GSK3174998	Plus pembrolizumab	NCT02528357	I	Advanced solid tumors	Completed
CD40	CP-870893	Plus tremelimumab or durvalumab	NCT01103635	I	Metastatic melanoma	Completed
	APX005M	Plus nivolumab and cabiralizumab	NCT03502330	I	Advanced melanoma	Recruiting
		Plus nivolumab	NCT03123783	I/II	Metastatic melanoma	Completed
		Plus pembrolizumab	NCT02706353	I/II	Metastatic melanoma	Recruiting
	JNJ-64457107/ADC-1013	monotherapy	NCT02829099	I	Advanced tumors	Active, not recruiting
	CDX-1140	Monotherapy	NCT03329950	I	Advanced tumors	Recruiting
GITR	TRX518	Plus gemcitabine, pembrolizumab, or nivolumab	NCT02628574	I	Advanced solid tumors	Completed
	GWN323	Plus spartalizumab	NCT02740270	I	Advanced solid tumors and lymphomas	Completed
	MK-4166	Plus pembrolizumab	NCT02132754	I	Advanced solid tumors	Completed
IDO1	Epacadostat	Plus pembrolizumab	NCT02752074	III	Unresectable/metastatic melanoma	Completed
IL-2	NKTR-214	Plus nivolumab	NCT03635983	III	Previously untreated inoperable or metastatic melanoma	Recruiting
IL-6	Tocilizumab	Plus nivolumab and ipilimumab	NCT03999749	II	Unresectable/metastatic melanoma	Recruiting
TNF-a	Inflimab/certolizumab	Plus nivolumab and ipilimumab	NCT03293784	I	Advanced melanoma	Recruiting
Oncolytic virus	T-VEC	Plus nivolumab	NCT04330430	II	Resectable early metastatic melanoma with injectable disease (NIVEC)	Recruiting
		Plus ipilimumab	NCT01740297	I/II	Advanced/unresectable melanoma	Completed
ACT	Lifileucel	Monotherapy	NCT02360579	II	Metastatic melanoma	Active, not recruiting

#### LAG-3

Lymphocyte activation gene-3 (LAG-3) is a T cells-associated inhibitor receptor that co-expressed with PD-1 on anergic or exhaustion T cells.^632^ Preclinical studies have shown that LAG-3 and PD-1 blockades synergistically stimulate T cell responses and decrease tumor burden in murine model.^633–635^ There are two LAG-3 inhibitors in clinical trials, IMP-321 and relatlimab (BMS-986016). The latest results of a phase III clinical trials combining relatlimab and nivolumab have demonstrated an improved antitumor activity in patients who showed tumor progression on PD-1 treatment, with a prominently prolonged median PFS versus nivolumab monotherapy (10.1 months vs 4.6 months) companied with higher incidence of treatment-related adverse events in the treatment of advanced melanoma.^636,637^ Clinical trials aiming to evaluate the safety and efficacy of relatlimab plus nivolumab are recruiting melanoma patients whose disease progressed on PD-1 monotherapy or naive to prior immunotherapy, as well as relatlimab in uveal melanoma.^638^

#### TIM-3

T cell immunoglobulin and mucin domain-containing protein 3 (TIM-3), with multiple ligands including galectin-9, high mobility group box 1 (HMGB1), and carcinoembryonic antigen-related cell-adhesion molecule 1 (CEACAM-1), functions as a co-inhibitory receptor on dysfunctional T cells.^639^ Co-inhibition of PD-1 and TIM-3 has demonstrated antitumor activity in preclinical studies,^640^ which leads to the development of TIM-3 blockades for clinical application. Phase I/II clinical trials have been initiated with the single use of TIM-3 antibody or combination with PD-1 blockade in melanoma. TIM-3 antibodies in clinical study mainly contain sabatolimab (MBG453), TSR-022, INCAGN02390, and LY3321367. While no response was seen with sabatolimab in advanced solid tumor including melanoma, patients undergoing the treatment of sabatolimab plus PD-1 antibody exhibit better response signs like elevated expression of immune markers.^641,642^ LY3321367 has demonstrated promising antitumor activity in single use or in combination therapies with PD-L1 antibody for the treatment of advanced cancers, with 68.2% and 88.2% response rate, respectively (NCT03099109). Studies on a bispecific antibody targeting both TIM-3 and PD-1 are also recruiting patients with advanced tumors including melanoma.

#### TIGIT

T cell immunoglobulin and ITIM domain (TIGIT), a promising new target for cancer immunotherapy, is upregulated mainly on activated T cells and NK cells as a co-inhibitory receptor.^643^ TIGIT impedes T cell and NK cell antitumor activities through the competing with CD226 binding to CD155 and CD122, two ligands on the surface of melanoma cells and APCs.^644–647^ Despite numerous clinical studies on TIGIT blockades, promising clinical results were merely seen with dual therapy of PD-1 blockades and only one TIGIT inhibitor, vibostolimab, for the treatment of advanced melanoma or PD-1-refractory melanoma.

T cells and NK cells express several cell surface co-stimulatory receptors which belong to TNFR family that induce the effector function of T cells and NK cells in tumor environment.^648^ Members of TNFR family including CD137, OX40, GITR, CD40, and CD27 have long been considered as viable immunotherapy targets.

CD137 (4-1BB), induced upon TCR stimulation, demonstrates co-stimulatory activities through boosting T cell proliferation, facilitating memory differentiation, and enhancing effector functions of both T cells and NK cells once binding to its ligand CD137L expressed on APCs.^649^ Although agonistic CD137 antibodies alone could not reinvigorate antitumor immunity against melanoma, CD137 agonists synergistically suppress melanoma with chemotherapy, radiotherapy, and other immunotherapy modalities including adoptive T cell therapy, ICBs, virotherapy and vaccines in mouse model and in vitro.^650–660^ However, clinical trials of the first agonistic CD137 antibody, urelumab (BMS-663513), have been hampered for severe liver toxicity in the treatment of advanced melanoma.^661^ Clinical trials of combinatorial regimen with urelumab and nivolumab are underway against melanoma. A phase Ib clinical study of utoliumab (CD137 agonist) plus pembrolizumab revealed improved safety but no synergic effects in advanced solid tumors.^662^ Combinatorial approaches of either optimized (LVGN6051) or lower dose (urelumab) of CD137 agonist and other immune modulators are also ongoing.^663^ Bispecific antibody (INBRX-105) targeting PD-L1 and 4-1BB also simultaneously enter the clinical use with tolerable safety and improved efficacy profiles.

#### OX40

OX40 (CD134) that belongs to TNFR superfamily 4 is mainly expressed on CD4^+^ T cells, CD8^+^ T cells, neutrophils, and NK cells driven by TCR-engaged activation.^664^ OX40 agonists promoted effector T cell expansion and survival, as well as depleted tumor-infiltrating Tregs.^665^ Preclinical studies have revealed that MEDI6383, a human OX40 ligand fusion protein, has the potential to boost antitumor immunity in human cancers,^666,667^ and clinical studies to evaluate the efficacy and safety of MEDI6383 is ongoing.^668^ Another CD134 agonist, MEDI0562, in combination with durvalumab or tremelimumab has demonstrated tolerable safety and clinical benefits, with median overall survival of 17.4 and 11.9 months for MEDI0562 plus durvalumab and MEDI0562 plus tremelimumab, respectively, in the treatment of advanced solid tumors.^669,670^ Combinatorial regimens of PF-8600 (OX40 agonist) and utomilumab (4-1BB agonist) has demonstrated a tolerable safety profile and clinical benefits with 70% melanoma patients achieving stable disease.^671^ A phase I dose-escalation trial of INBRX-106, a novel hexavalent OX40 agonist, has revealed the safety profile and clinical benefits in patients with a range of cancer types. Clinical trials of INBRX-106 with or without pembrolizumab are recruiting for the treatment of advanced tumors including melanoma. MOXR0916, an agonist monoclonal antibody targeting OX40, is under clinical investigations in combination with atezolizumab.^672^ GSK3174998 and BMS-986178, both of which are humanized IgG1 agonistic OX40 monoclonal antibodies, have very modest combination therapeutic activities with ICBs against advanced solid tumors, although with a tolerable safety profile.^673,674^ Other forms of immunotherapy modalities targeting OX40L are being developed to overcome the low efficacy of OX40 agonists. SL-279252, a first-in-class agonist redirected checkpoint fusion protein including PD-1 and OX40L, the clinical trial of which is also ongoing. DNX-2440, a tumor-selective conditionally replicative oncolytic adenovirus expressing OX40L is being developed for the treatment of cancers with liver metastasis.

#### GITR

Similar to CD137 and CD134, glucocorticoid-induced TNFR related protein (GITR) modulates T cell activation by providing a co-stimulatory signaling. However, clinical trials of GITR agonists, TRX518, AMG228, MK-1248, MK-4166, BMS986156, MEDI18730, and GWN323 showed limited antitumor activities in monotherapy, although with tolerable safety profiles.^675–681^ These studies have implicated that GITR agonists alone could not reactivate the cytolytic function of T cells in tumor environment, and combinatorial approaches of GITR agonism and other immunomodulatory therapies are of great interest. Responses were observed in ICBs-naive melanoma patients (ORR, 62%) with the combination of MK-4166 and Pembrolizumab.^677^

#### CD40

CD40, also known as TNFRSF5, is mainly expressed on DCs, B cells, and macrophages whereas its ligand, CD154, is transiently expressed by activated T cells.^682^ CD40 agonists could lead to T cell activation through increased antigen presentation and elevated level of critical T cell stimulatory cytokines.^683^ CP-870893, a fully human IgG2 monoclonal antibody against CD40, exhibited well tolerable safety profile and clinical benefits. 27% of melanoma patients had objective partial responses with the treatment of CP-870893, which, however, was not reproducible.^684,685^ Combined regimens containing CP- 870893 with tremelimumab or the immune stimulant, oncovir poly IC:LC, along with a melanoma vaccine, NY-ESO-1/gp100, are under evaluation in a phase I trial in patients with melanoma. Another CD40 agonist, APX005M and the inhibitor of another macrophage polarizing regulator CSF1R, cabiraluzumab with or without nivolumab demonstrated tolerable safety profile, warranting further investigations into the optimization of the dosing and selection of patients.^686^ The investigations on the combinations of APX005M and nivolumab or pembrolizumab are still ongoing. Other CD40 agonists entering the clinic including SEA-CD40, ADC-1013, and CDX-1140 are being tested for single use or in combination with either chemotherapy, vaccines, or ICBs in early clinical trials in patients with advanced melanoma.^687,688^

#### IDO1

Indoleamine 2,3-dioxygenase 1 (IDO1) is an intracellular IFNγ-inducible enzyme that converts tryptophan to kynurenine, which leads to a suppressed tumor environment through impairing T cell proliferation and activity due to amino acid deficiency and promoting the differentiation of Tregs.^682^ IDO1 is overexpressed in a variety types of cancers including melanoma.^689^ Low level of IDO1 in melanoma metastasis is associated with improved overall survival and could predict the outcome of metastatic melanoma patients with immunotherapies.^690–692^ Preclinical studies have implicated that pharmacological inhibition of IDO1 enhanced T cell response and impeded tumor growth.^693–698^ Epacadostat, a selective reversible IDO1 inhibitor, has no effect as monotherapy or in combination with pembrolizumab for the treatment of multiple types of solid tumors. Although epacadostat achieved a favorable response rate of 55% in combination with pembrolizumab for the use in patients with solid tumors from a phase I/II trial, which led to a phase III study to evaluate the efficacy of epacadostat in 706 unresectable melanoma patients, the results from the larger trial revealed that epacadostat plus pembrolizumab did not provide an improved overall survival or progression-free survival.^699,700^ Due to the negative results of the trial, multiple clinical trials of epacadostat were halted. Another IDO1 inhibitor, BMS-986205, in combination with ipilimumab showed modest clinical benefit with an ORR of 26%.^701^ Clinical trials of other two IDO1 inhibitors, indoximod and navoximod demonstrated tolerable safety and antitumor efficacy worthy of further evaluations in advanced melanoma.^702,703^

Agents targeting other immune inhibitor receptors, such as B7-H3, BTLA, and VISTA, stimulatory receptor including CD27 and CD70 and checkpoints on NK cells containing NKG2A and KIR family are also under evaluation for further clinical application in melanoma patients. Besides, other immunomodulatory molecules, TLR, TNFα and IL-10 are also appealing for potential combination with ICBs to treat melanoma.^704,705^

## Conclusion and future perspectives

Melanoma is the most lethal skin cancer that results from the malignant transformation of melanocytes. Intense UVR, multiple moles, family history, and fair skin are the main risk factors associated with increased incidence of melanoma. In the past few decades, the therapeutic approaches have gained revolutionary advances due to a deeper understanding of the molecular mechanisms underlying melanoma pathogenesis. In particular, the wide application of targeted therapy and immunotherapy has substantially improved the 5-year-survival of patients with advanced melanoma from <10% to around 30%. Nevertheless, the prognosis of patients remains suboptimal because of the low response rate and frequent occurrence of treatment resistance to currently available therapies. Therefore, it is of necessity to obtain a more comprehensive understanding of the mechanisms driving distinct aspects of melanoma biology, including mutated driver genes, transcriptional regulation of tumor biology, dysregulated epigenetic modifications, metabolic reprogramming, metastasis-associated modifiers, and tumor-promoting inflammatory signals and angiogenesis (Table 4), which might bring about more promising and innovative therapeutic strategies.

**Table 4:** Summary of signaling pathways and potential therapeutic targets in the present review

Mutated driver genes	
MAPK pathway	BRAF, NRAS, MEK, ERK, KIT, STK19
Cell-cycle regulation pathway	CDK4/6, MDM2, Cyclin D1, Rb
AKT pathway	PI3K, Akt
Pigmentation-related pathway	MC1R, Tyrp1, Pax3, Ednrb, MITF, SOX10
Other pathways	GNAQ/GNA11, Notch2, β-catenin, ARID1B, ARID2, TERT
Key transcriptional pathways	
SOX10 pathway	Sox10, MITF, lncRNA SAMMSON, FOXD3, Rab7
MITF pathway	MITF, Bcl2, Bcl2a1, ML-IAP, HIF1α, c-Met, APE1, p21, BRAC1, SCD
Notch and Wnt pathways	Notch, β-catenin, LEF-1
Epigenetic regulation-related pathways	
DNA methylation	DNMTs, TET family, IDH2
Histone acetylation and methylation	LSD family, HDAC family, EP300, SETDB1, Dot1L, EZH2, JMJD2C
Non-coding RNA and m^6^A RNA methylation	SAMMSON, FTO, ALKBH5, METTL3/14, YTHDF1
Metabolic reprogramming	
Aerobic glycolysis	HIF1α, MYC, Glut 1, Glut 3, HK2, PFKFB2, MCT-4, HMGCL, Oct-1
Oxidative phosphorylation	MITF, PGC1α, lncRNA SAMMSON, p32
Lipid metabolism	ACLY, ACC, FASN, ACS, ACAT2, HMGCS, HMGCR, MVK, SREBP-1, SREBP-2, CD36
Autophagy	Atg5, Twist1, p62, Atg7, SIRT6, Akt, IGF, miR-23a, TFEB, UPR pathway, RIPK1
Amino acid metabolism	PHGDH, GLS2, GOT1, BCAT1/2, BCKDH, DBT
Key signal pathways in tumor metastasis	
EMT process	N-cadherin, vimentin, α-SMA, SOX2, Snail, Slug, Twist, ZEB1/2, NF-κB, FRA1
CAM	integrin, cadherin, IgSF, connexin, mucin, ILK
Exosomes	Rab27A, let-7i, miR-106b-5p, miR-125b-5p,
Signal pathways in oncogenic inflammation and angiogenesis	
Inflammatory factors	TNFα, IFN-γ, interleukins, JAK-STAT, NF-κB, NALP3 inflammasome, c-Jun
Angiogenesis	VEGF-A, bFGF, PlGF, Ang, IL-8, PDGF, VEGFR, NOS, FAK

The low response rate to immunotherapy and inevitable establishment of resistance to targeted therapy and immunotherapy significantly hinder the treatment efficacy. Recently, more and more investigations have emphasized elucidating the underlying mechanisms, so as to develop novel combined therapy to improve the patients’ outcome. Of note, immunotherapy has been the leading edge of melanoma treatment, with more and more targets revealing encouraging translational potential not only as a single agent but also in combined therapy. Severe adverse effects should be noted and overcome during the process of immunotherapy innovations. More importantly, the heterogeneity of melanomas needs to be taken into consideration and this characteristic determines the fact that a single molecular or pathway might not yield more therapeutic choices. More attention needs to be paid on the study of minority subgroup of melanoma, for example, acral melanoma, in which current therapies display poor effects. The path to clinical translation of novel therapeutic approaches is still in demand of great efforts in the future.
